# Non-coding RNAs/DNMT3B axis in human cancers: from pathogenesis to clinical significance

**DOI:** 10.1186/s12967-023-04510-y

**Published:** 2023-09-13

**Authors:** Chunjie Huang, Paniz Azizi, Masoud Vazirzadeh, Seyed Mohsen Aghaei-Zarch, Fatemehsadat Aghaei-Zarch, Jalaledin Ghanavi, Poopak Farnia

**Affiliations:** 1 Institute of Reproductive Medicine, School of Medicine, Nantong University, Nantong, 226001 China; 2 Department of Psychological and Brain Science, Program in Neuroscience, Indiana University Bloomington, Bloomington, IN USA; 3 Department of Cell and Molecular Biology and Microbiology, Faculty of Biological Science and Technology, University of Isfahan, Isfahan, Iran; 4 Department of Medical Genetics, School of Medicine, Shahid Beheshti University of Medical Sciences, Tehran, Iran; 5 School of Medicine, Kerman University of Medical Sciences, Kerman, Iran; 6 Mycobacteriology Research Center, National Research Institute of Tuberculosis and Lung Disease, Shahid Beheshti University of Medical Sciences, Tehran, Iran

**Keywords:** Noncoding RNAs, Cancer, DNMT3B

## Abstract

Cancer is a complex disease with many contributing factors, and researchers have gained extensive knowledge that has helped them understand the diverse and varied nature of cancer. The altered patterns of DNA methylation found in numerous types of cancer imply that they may play a part in the disease’s progression. The human cancer condition involves dysregulation of the DNA methyltransferase 3 beta (DNMT3B) gene, a prominent de novo DNA methyltransferase, and its abnormal behavior serves as an indicator for tumor prognosis and staging. The expression of non-coding RNAs (ncRNAs), which include microRNAs (miRNA), long non-coding RNAs (lncRNAs), and circular RNAs (circRNAs), is critical in controlling targeted gene expression and protein translation and their dysregulation correlates with the onset of tumors. NcRNAs dysregulation of is a critical factor that influences the modulation of several cellular characteristics in cancerous cells. These characteristics include but are not limited to, drug responsiveness, angiogenesis, metastasis, apoptosis, proliferation, and properties of tumor stem cell. The reciprocal regulation of ncRNAs and DNMT3B can act in synergy to influence the destiny of tumor cells. Thus, a critical avenue for advancing cancer prevention and treatment is an inquiry into the interplay between DNMT3B and ncRNAs. In this review, we present a comprehensive overview of the ncRNAs/DNMT3B axis in cancer pathogenesis. This brings about valuable insights into the intricate mechanisms of tumorigenesis and provides a foundation for developing effective therapeutic interventions.

## Introduction

The prevalence of cancer is a significant contributor to global mortality. It is projected that 8.2 million individuals worldwide died due to cancer in 2012, and the United States is predicted to witness over six hundred thousand cancer-related deaths in 2020 alone. Cancer is a complex and heterogeneous ailment in which neoplastic cells exhibit shared mechanisms of tumorigenesis, progression, and invasive potential. Over the course of 10 years, researchers have identified eight hallmarks that bestow cancer cells with the ability to endure, propagate, and metastasize. The identified hallmarks of cancer include the potential of neoplastic cells to maintain self-sustaining signaling for proliferation, elude destruction by the immune system, disrupt cellular energetics, enable perpetual replication, trigger metastasis and invasion, stimulate angiogenesis, evade inhibitory signals that restrain growth, and resist programmed cell death. Furthermore, the two traits of “tumor-promoting inflammation” and “genome instability and mutation” have been included as contributing factors towards the acquisition of hallmarks. A recent hypothesis suggests that irregularities in epigenetic processes and chromatin structures may result in substantial oncogenic advantages, consequently fulfilling all of the identified hallmarks of cancer. The two most frequently observed forms of mammalian epigenetic modifications are histone acetylation/methylation and DNA methylation. Of these, DNA methylation is a prominent epigenetic mechanism that is implicated in various stages of evolution and in the progression of cancer. DNA methylation refers to the process of adding a methyl group to the C-5 position of the cytosine ring of DNA through covalent linkage. In the context of human cancer, there is a disruption in the typical patterns of DNA methylation. Specifically, the level of DNA methylation is generally higher in cancer cells compared to normal cells, and there is an increased occurrence of methylation in CpG islands associated with tumor suppressor genes and DNA repair mechanisms. The process of DNA methylation is facilitated by a group of enzymes known as DNA methyltranferases (DNMTs). Within the DNMT family, there exist three distinct enzymes: DNMT1, which is accountable for preserving existing methylation patterns following DNA replication, and DNMT3A and DNMT3B, both de novo methyltransferases that play a crucial role in establishing methylation during imprinting and development. A recent experimental study has revealed the significance of DNMT3B dysregulation in carcinogenesis. In this regard, Peralta-Arrietathis et al. conducted an investigation to examine the impact of DNMT3B overexpression on global gene expression and the methylation of specific genes in HaCaT cells, with the aim of identifying potential targets of DNMT3B. The study disclosed that overexpressing DNMT3B in HaCaT cells resulted in the modulation of genes associated with cancer. Specifically, it led to the downregulation of 151 genes containing CpG islands and the downregulation of the VAV3 gene via promoter methylation. Therefore, their findings emphasize DNMT3B significance in both gene expression and the development of human cancer [1]. Current in-depth research highlights the importance of non-coding RNA molecules in directing DNMT3B function, while also contributing to our comprehension of how ncRNA regulates DNMT3B. This emphasizes the extensive participation of ncRNAs and their interactions with key epigenetic modifiers, including DNMT3A, in controlling the expression of numerous target genes. Thereby, in this review, we compiled literature on ncRNAs associated with DNMT3B in human cancer, along with the potential underlying mechanisms linking them, which play a role in all stages of human cancer. Based on them, it is expected that an overwhelming number of demands for the diagnosis and treatment of human cancer will continue to emerge at an increasing rate.

### Noncoding RNAs: from biology to functioning in epigenetics

It is noteworthy that mRNA constitutes only a small fraction (about 2%) of the extensively transcribed human genome, whereas the noncoding majority was previously deemed as insignificant. However, it is now acknowledged that this noncoding portion plays a crucial role in cellular homeostasis and dysfunction through noncoding RNA genes, interspersed nuclear elements, introns, and regulatory DNA sequences. NcRNAs, such as microRNA, long-noncoding RNA, and circular RNA, constitute a significant proportion of the human transcriptome and are known to exert crucial functions in diverse pathophysiological processes [2].

#### MiRNA

miRNAs are a subset of small noncoding RNAs that are found in both plant and animal cells and typically consist of 18–25 nucleotides [3]. The initial discovery of miRNAs in *Caenorhabditis elegans* can be traced back to Lee and colleagues’ work in 1993. Subsequently, in 2001, small regulatory RNAs were identified in plants and mammals, which were eventually classified as miRNA. MiRNAs originate from hairpin-shaped transcripts, which are typically produced by RNA polymerase II. This transcription process yields extended primary transcripts, or pri-miRNAs, much like the majority of genes present in the genome. The initial transcripts undergo processing via a Drosha complex, which comprises the co-factor DGCR8 and an RNAse type III enzyme, leading to the formation of pre-miRNAs that are around 70–100 nucleotides in length [4]. Additionally, an alternative pathway that operates independently of Drosha but relies on splicing machinery has been characterized in various species. Pre-microRNAs undergo subsequent processing mediated by RNAse III Dicer, which generates a double-stranded RNA molecule consisting of approximately 22 nucleotides. This RNA duplex encompasses the mature microRNA strand, also referred to as miRNA-5p, in addition to its complementary strand known as miRNA or miRNA-3p [5, 6]. Typically, miRNAs undergo degradation but they can also function as miRNAs. Following maturation, the miRNA forms a single strand and can regulate the expression of protein-coding mRNAs by binding to partially complementary regions commonly found at the 3′-untranslated region (3′-UTR) of the target transcript [7]. An increasing body of evidence suggests that miRNAs and epigenetic machinery are reciprocally regulated. MiRNAs have the ability to target genes that regulate epigenetic pathways. Multiple miRNAs are capable of regulating chromatin structure by controlling DNA modifier molecules, such as DNMT3B. The expression of these miRNAs, referred to as epi-miRNAs, has significant implications for the epigenetic regulation of various cellular pathways and processes. In this regard, the potential involvement of miR-29c in the fibrotic processes associated with biliary atresia was examined by Wang and colleagues. Using a luciferase reporter assay, they revealed that miR-29c could selectively bind to the 3′-UTR of DNMT3B, thereby suppressing its expression [8]. Importantly, there is an increasing body of evidence indicating that mature miRNAs, in conjunction with the components of RNA-induced silencing complex (RISC), are capable of translocating to the nucleus and exerting direct regulatory effects on gene and ncRNA expression at the genomic level. Such regulatory effects may be attributed to either induction or suppression of transcription [9]. In this context, Mauro and colleagues have demonstrated that the canonical Wnt pathway inactivation triggers the nuclear translocation of miR-133a in cardiac cells. They have also revealed that the miR-133a/AGO2 complex present in the nucleus binds to a complementary target site for miR-133a within the promoter region of the de novo DNMT3B gene, resulting in its transcriptional suppression. This process is facilitated by DNMT3B itself. Altogether, the data presented indicate an atypical function of miR-133a, which, upon its translocation to the nucleus, induces epigenetic suppression of the target gene DNMT3B through a negative self-regulatory feedback loop involving DNMT3B [10]. Furthermore, cytosine within the dinucleotide sequence of CpG islands is the primary site for DNA methylation. The silencing of miRNA genes and their tumor suppressor properties are attributed to the CpG islands hypermethylation located in the promoter region. Typically, the methylation process of CpG islands located within miRNA promoters leads to the binding of methylation binding proteins with DNA. This binding subsequently leads to the inhibition of transcription factors and RNA polymerase from binding with DNA, resulting in miRNA gene expression repression. On the other hand, CpG islands hypomethylation led to gene expression activation, thereby promoting cancer progression [11].

#### LncRNA

The lncRNA conventional definition entails the presence of a transcript that exceeds 200 nucleotides and lacks the capacity to encode proteins [12]. Nonetheless, restricting classification of lncRNAs solely based on their length may be overly limited and rigid. Based on their genomic location, morphology, sequence, structure, and functional characteristics, various groups of lncRNAs can be distinguished. There are also commonalities between lncRNAs and protein-coding mRNAs, such as the presence of a 3′ poly (A) tail, a 5′ cap, and intron–exon structures. Nevertheless, certain lncRNAs, which are highly prevalent, appear to deviate from conventional paradigms by either not undergoing processing or being processed in a noncanonical manner [13]. LncRNAs possess diverse capabilities, as their functional repertoire is contingent upon their cellular localization and intricate interactions with DNA, RNA, and proteins. Leveraging these attributes, lncRNAs can govern chromatin dynamics, modulate the assembly and operation of non-membrane-bound nucleosomes, elicit alterations in the stability and translational efficiency of cytoplasmic mRNA, and perturb signal transduction pathways. Notably, these multifaceted mechanisms collectively contribute to the modulation of gene expression patterns in various biological and pathophysiological contexts, encompassing cancer [14]. Furthermore, lncRNAs exhibit the capacity to serve as scaffolds or guides, and can impede the binding of RNAs and proteins, including DNMT3B. The interactions between DNMTs, and lncRNAs display a diverse and mutually influential nature. For example, lncRNAs possess the ability to enlist DNMTs, thereby directing them towards the promoters of target genes and exerting regulatory control over their expression patterns. Conversely, alterations in the methylation status of specific lncRNA gene promoters can impact the expression levels of both the lncRNAs themselves and the downstream genes under their regulation. In this context, the research conducted by Deng et al. sought to explore the potential involvement of lncRNA Growth arrest-specific transcript 5 (GAS5) in the advancement of ischemic stroke by examining its regulatory influence on the methylation of mitogen-activated protein kinase 4 (MAP4K4). Through a comprehensive set of experimental methodologies, they successfully corroborated that GAS5 elicited a considerable impact on the methylation status of MAP4K4, resulting in a noteworthy downregulation of MAP4K4 expression by virtue of its recruitment of DNMT3B. Additionally, their findings underscore the potential therapeutic significance of GAS5 inhibition in mitigating neuronal apoptosis and ameliorating neurological deficits associated with ischemic stroke. This effect is achieved through the suppression of DNMT3B-mediated methylation of MAP4K4 [15]. In this manner, lncRNAs exhibit the capacity to serve as both broad-spectrum and targeted regulators of DNA methylation by modulating the expression or recruitment of DNMT3A to specific genes.

#### CircRNA

**Fig. 1:**
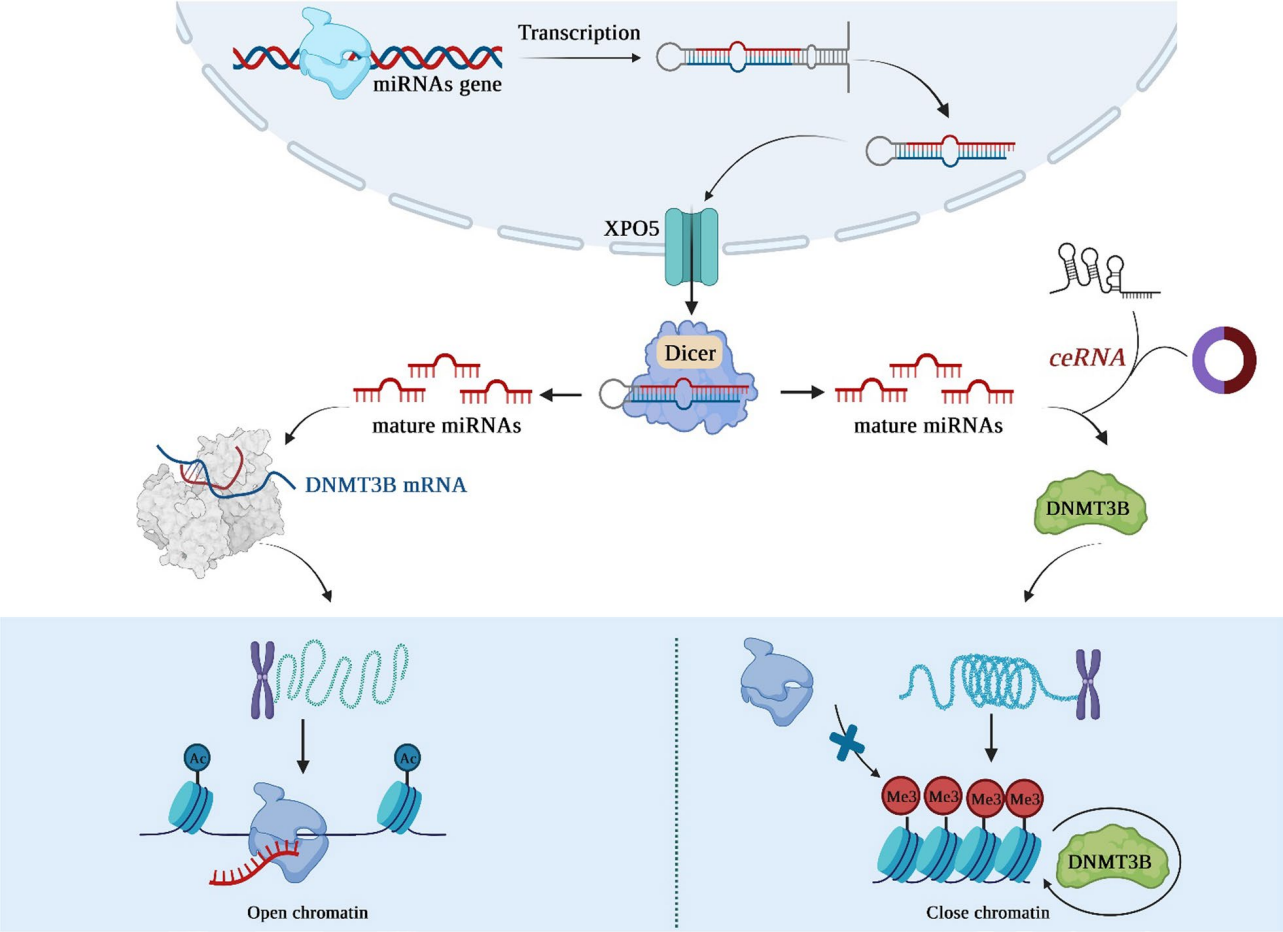
A schematic representation of DNMTs regulation via non-coding RNAs

CircRNAs are commonly classified as ncRNAs; however, emerging evidence substantiates the ability of certain circRNAs to encode functional proteins. Distinguished from conventional linear RNAs, circRNAs possess distinctive circular structures with closed loops, lacking both 5′ caps and 3′ tails. The majority of identified circRNAs originate from RNA-polymerase II-mediated transcription of protein-coding genes [16]. The unique circular configuration of circRNAs confers resistance to cellular exonucleases, endowing them with exceptional stability as RNA molecules when compared to canonical mRNA transcripts. Hence, circRNAs have the potential to serve as disease biomarkers and play a role in monitoring treatment effectiveness, as well as serving as targets for novel therapeutic interventions. Despite the extensive cataloging of thousands of circRNAs, the functional roles of only a limited subset have been comprehensively characterized. One notable example among circRNAs is circCDR1as, which stands as the most prevalent circRNA in mammals. In 2013, the pioneering discovery regarding circRNAs revealed their capacity to function as miRNA sponges. Since then, the most extensively established role of circRNAs has been associated with their ability to sequester miRNAs and proteins. In this regard, ciRS-7 exhibits over 70 binding sites that are conserved for miR-7, and it has the ability to interact with the Argonaute (AGO) protein. This interaction between circRNAs and miRNAs facilitates the sequestration of miRNAs, enabling the translational machinery to specifically bind to target mRNA molecules such as DNMT3B. Consequently, this circRNA-miRNA sponge formation leads to the derepression of genes. In conclusion, circRNAs by modulating DNMT3B activity have the potential to assume a progressively significant role in the epigenetic regulation governing the initiation and advancement of human cancer (Fig. 1).

### DNMT3B: from biology to functioning in human cancer

**Fig. 2:**
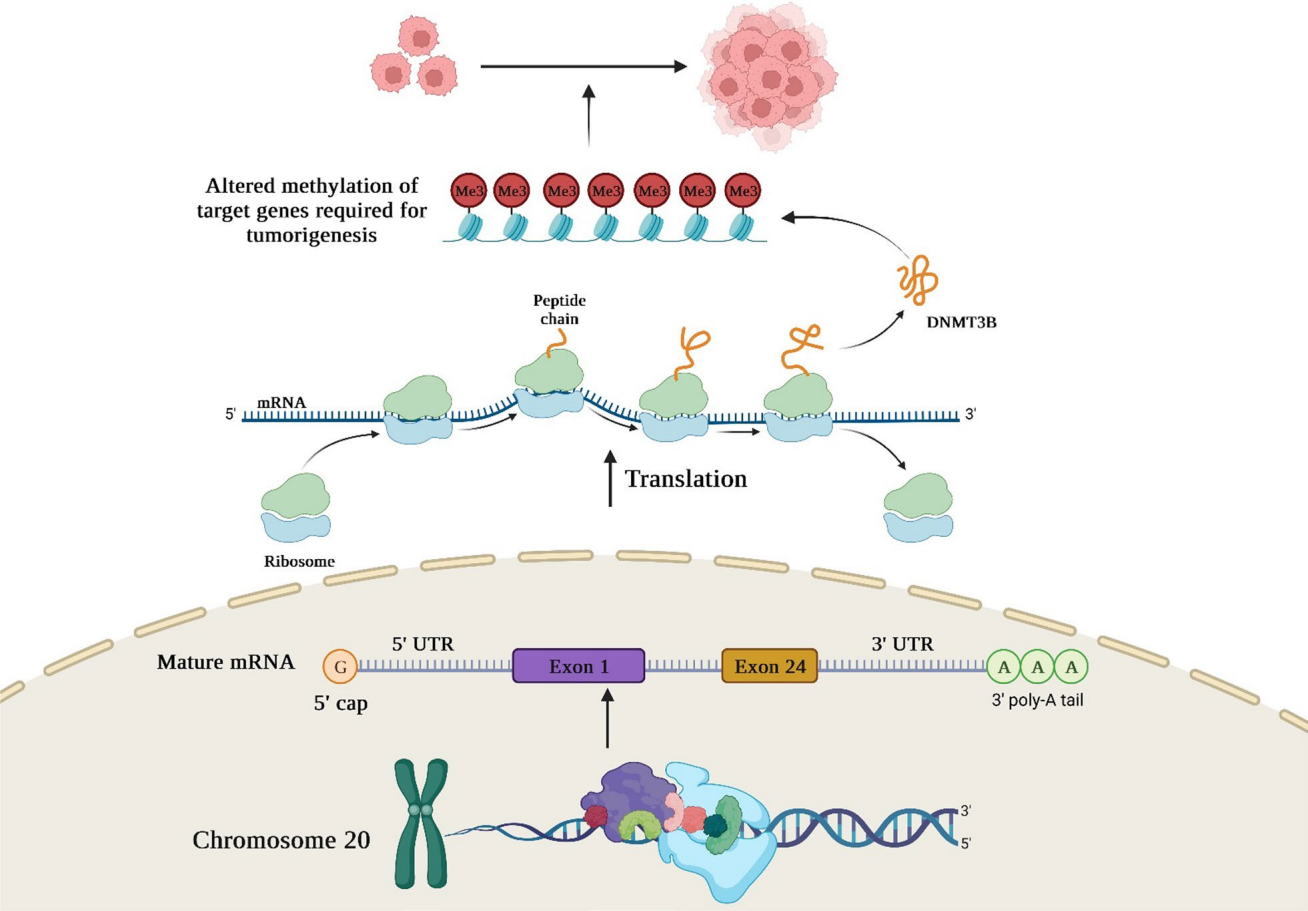
A schematic representation of DNMT3B location and expression

DNMT3B functions as a DNA-(cytosine C5)-methyltransferase, playing a pivotal role in the establishment of methylation patterns within DNA. Working in collaboration with DNMT3A, it operates through the de novo methylation pathway to carry out this essential epigenetic process. The identification of human DNMT3B, a gene that exhibits significant homology to the mouse Dnmt3b gene, was initially accomplished through a thorough investigation of databases in the year 1998. Subsequent to this, numerous investigations have successfully cloned the cDNA sequence of the aforementioned subject, ascertained its specific position on the chromosomal locus 20q11.2, and revealed its extensive expression in various normal human tissues, alongside its marked upregulation in carcinogenesis. DNMT3B comprises a catalytic domain located at the C-terminus, acknowledged as the pivotal site of enzymatic activity. Additionally, it encompasses a regulatory domain positioned at the N-terminus, housing two functionally conserved domains. These regulatory domains play a crucial role in facilitating the precise localization of chromatin and governing their functional processes. The functional domain known as PWWP, is recognized for its distinctive Pro-Trp-Trp-Pro core sequence motif. Another noteworthy functional domain found in the N-terminal region is ADD, notable for its high abundance of Cysteine residues. In the C-terminal catalytic domains, there exists a set of 10 distinctive motifs that are indicative of DNA-(cytosine C5)-methylation functionality. DNMT3B, in conjunction with DNMT3L, engages with this particular domain to augment the process of de novo DNA methylation. The precise localization of DNMT3B at specific genomic sites is intricately controlled through the interplay between interactions involving de novo methyltransferase and chromatin remodeling complexes, as well as the influence of transcription factors and histone modifications. Previous studies have indicated that DNMT3B exhibits a combination of unique and shared functionalities in relation to its paralog, DNMT3A. Notably, these two enzymes possess a notable degree of similarity within their catalytic domains. The distinctive binding characteristic could potentially be attributed to the N-terminal region of the protein. Specifically, the ADD domain of DNMT3B facilitates its ability to recognize and selectively bind histone 3 tails that lack methylation at lysine 4, thereby impeding its binding to active TSS. Recent studies have brought attention to the crucial role played by the PWWP domain in the recruitment of DNMT3B protein to gene bodies. This recruitment is mediated through the specific recognition of histone 3 lysine 36 trimethylation (H3K36me3) enrichment within those genomic regions. The association between DNMT3B and H3K36me3 was also detected in human epidermal stem cells, wherein DNMT3B interacts with the genomic regions corresponding to actively transcribed cell-type-specific enhancers. This interaction facilitates the enzymatic activity of DNMT3B, leading to the induction of hypermethylation within these enhancer elements. Accumulating evidence has provided significant support for the involvement of DNA hypermethylation/hypomethylation, dysregulation of DNMT3B, and chromatin remodeling in the processes underlying cancer initiation, and development. In this regard, Kim et al. conducted an investigation into the clinical and prognostic significance of DNMT3B in the context of hepatocellular carcinoma (HCC). Their findings revealed that increased expression of DNMT3B in HCC patients was indicative of reduced overall survival rates, while HCC cases characterized by lower levels of DNMT3B expression exhibited a shorter duration of disease-free survival. The researchers postulated that the expression of DNMT3B could potentially play a crucial role in determining the prognosis of HCC [17]. Furthermore, So et al. conducted a study aimed at elucidating the mechanisms underlying the selection and evolution of cancer cells leading to the establishment of distant metastatic colonies. Their investigation initially revealed the presence of heterogeneous expression patterns of DNMT3B in primary tumors of both humans and mice. Through their mechanistic analysis, they revealed that clonal cells exhibiting elevated DNMT3B levels demonstrated increased expression of vimentin (VIM) and exhibited augmented capacity for epithelial-to-mesenchymal transition. Furthermore, VIM deletion mitigated the metastatic phenotype observed in DNMT3BH cells. Furthermore, in preclinical mouse models where primary tumors were surgically excised, they observed that perioperative intervention targeting DNMT3B, in conjunction with chemotherapy, significantly suppressed the recurrence of tumors and the formation of metastases. In this manner, they have identified the role of DNMT3B-mediated transcription regulation as a significant factor contributing to tumor heterogeneity. Additionally, they have demonstrated that DNMT3B plays a critical role in tumor invasion, thereby highlighting its potential as a viable therapeutic target for addressing metastatic diseases [18]. In this manner, these findings indicated that DNMT3B dysregulation is linked to cancer development and progression (Fig. 2).

### Noncoding-RNA/DNMT3B axis in cancer pathogenesis

#### Gastrointestinal cancer

On a global scale, gastrointestinal malignancies encompass over 25% of all reported cancer cases and account for approximately one-third of cancer-related deaths. While significant advancements have been made in the screening methods for colorectal cancer, the prognosis for other forms of gastrointestinal malignancies remains generally unfavorable [19]. Despite the understanding that genetic and epigenetic changes play a crucial role in the progression of colorectal cancer (CRC), the specific molecular mechanisms governing the advancement of neoplasia in this context remain inadequately elucidated. Therefore, to comprehensively comprehend the molecular mechanisms involved in gastrointestinal cancer and to pinpoint specific target sites for interventional measures, it is imperative to assess prognosis and devise effective treatments for individuals afflicted with gastrointestinal malignancies [20].

##### MiRNAs/DNMT3B axis in colorectal cancer

MiRNAs have been associated with the progression of colorectal cancer (CRC), and these entities have recently been examined as prospective novel biomarkers for both the diagnosis and therapeutic intervention of CRC [21]. Yun et al. investigate the expression profile and functional significance of miR-432-5p in CRC along with its associated molecular mechanism. Their research findings based on analysis of the GSE136020 dataset indicated a downregulation of miR-432-5p in CRC. Subsequently, they identified CCND2 mRNA as a validated target of miR-432-5p. The researchers also revealed a notable downregulation of miR-432-5p, while observing abundant expression of CCND2 in both CRC tissues and cells. Their further experimentation unveiled that DNMT3B facilitated the process of DNA methylation specifically at the CpG Island associated with miR-432-5p, resulting in its expression inhibition. Furthermore, they observed that the introduction of a miR-432-5p mimic considerably impeded the development of primary CRC in mice, indicating its potential therapeutic efficacy as a tumor suppressor. Furthermore, it was discovered that the decrease in DNMT3B expression compromised the viability and invasiveness of CRC cells in vitro. It also impeded cell cycle progression in these cells. In vivo experiments using nude mice demonstrated that downregulation of DNMT3B hindered the growth of xenograft tumors and suppressed metastasis. Ultimately, the researchers concluded that when miR-432-5p was further downregulated or CCND2 was upregulated, the malignant characteristics of CRC cells were restored. Their findings substantiated that DNMT3B facilitated DNA methylation and subsequent downregulation of miR-432-5p as a means to promote CRC development by enhancing the expression of CCND2 [22]. Furthermore, To et al*.* aimed to elucidate the importance of efflux transporter ABCG2 downregulation in relation to both the initiation and progression of CRC. They successfully established that miR-203 directly targets the de novo DNA methyltransferase DNMT3B. Moreover, they revealed that the diminished expression of miR-203 in CRC led to the alleviation of DNMT3B repression, resulting in the methylation of the ABCG2 promoter and a significant decrease in ABCG2 expression observed in both colon cancer cell lines and patient-derived CRC specimens. In conclusion, the researchers determined that manipulating the newly discovered microRNA-methylation mechanism to restore ABCG2 function in precancerous cells could potentially serve as a promising strategy to effectively delay the progression of carcinogenesis [23]. Moreover, Shahmohamadnejad et al. conducted a study examining the impact of methylation, downregulation, and upregulation of miR-124, as well as its target gene DNMT3B, on the migration, and proliferation capabilities of a colorectal cell line. They methylation-specific PCR (MSP) analysis demonstrated pronounced hypermethylation within the promoter region of miR-124, consequently leading to a noteworthy decrease in its expression within tumor tissues. They also revealed an observed upregulation of miR-124 expression subsequent to treatment with 5-AZA-CdR. additionally, transfection of the Hct-116 cell line with miR-124 was found to result in reduced expression of DNMT3B, along with diminished cellular proliferation, and invasion capabilities within the HCT-116 cells. In summary, their findings provide evidence suggesting that miR-124 exerts suppressive effects on the proliferation, and invasion of colorectal cancer cells via DNMT3B downregulation [24].

##### MiRNAs/DNMT3B axis in esophageal cancer

Yang et al. conducted an investigation to examine the roles of miR-149 and its associated molecules in esophageal squamous cell carcinoma (ESCC). Their research revealed that miR-149 exhibited low expression levels, while DNMT3B and ring finger protein 2 (RNF2) displayed high expression levels within ESCC tumor samples. Through their in vitro and in vivo analyses, the researchers unveiled that the miR-149 upregulation effectively inhibited the invasiveness and proliferation of ESCC cells. They further revealed that DNMT3B exhibited binding affinity to the miR-149 promoter region, subsequently inducing methylation of its promoter and subsequent downregulation. Additionally, it was disclosed that miR-149 directly targeted RNF2 as part of its regulatory mechanism. Furthermore, the researchers observed that the overexpression of RNF2 counteracted the inhibitory impact exerted by miR-149 on the growth of ESCC cells. Moreover, RNF2 upregulation was found to activate the Wnt/β-catenin signaling pathway, thereby facilitating the progression of ESCC. In this manner, DNMT3B regulates the level of miR-149 by modifying the methylation status of the miR-149 promoter. At the same time, miR-149 inhibits the expression of RNF2 and deactivates the Wnt/β-catenin pathway in order to restrict the growth of ESCC cells [25]. Furthermore, Peng et al. explored miR-129-2-3p regulatory mechanism in the advancement of esophageal carcinoma (EC) cells. They revealed a significant downregulation of miR-129-2-3p expression in EC cell lines, whereas DNMT3B exhibited a markedly elevated expression level. The researchers made a significant discovery regarding the binding capability of miR-129-2-3p to DNMT3B. They via in vitro functional assays revealed that the miR-129-2-3p upregulation effectively suppressed the progression of EC cells. Interestingly, their findings also indicated that the inhibitory effect could be counteracted by the additional overexpression of DNMT3B. In this manner, miR-129-2-3p acts as a tumor suppressor in EC cells by targeting and inhibiting DNMT3B [26].

##### LINC00240/DNMT3B axis in gastrointestinal cancer

LINC00240, a recently identified lncRNA, has garnered attention due to its aberrant modulation in various tumor diseases. Substantial evidence supports its involvement in the regulation of tumor initiation and progression, particularly in gastric cancer (GC) [27]. Li et al. conducted an investigation to examine the biological implications of LINC00240 in GC tumorigenesis and elucidate its potential underlying mechanisms. Their research revealed elevated expression levels of LINC00240 in both GC tissues and cells, indicating its potential involvement in GC pathogenesis. In their study, the researchers observed that by downregulating LINC00240, they were able to effectively slow down cell proliferation, and migration in vitro. Furthermore, in vivo experiments demonstrated a significant suppression of GC cell tumorigenesis. Their Subsequent investigations revealed that LINC00240, a cytoplasmic lncRNA, possesses miRNA response elements of miR-124-3p and shares these elements with DNMT3B. As a result, it forms a functional axis termed LINC00240/miR-124-3p/DNMT3B, which provides insights into the underlying mechanisms involved in the functions of LINC00240. Overall, their study establishes LINC00240 as an oncogene that promotes GC tumorigenesis through the aforementioned LINC00240/miR-124-3p/DNMT3B axis [28].

##### Circ_0000467/DNMT3B axis in gastrointestinal cancer

Circ_0000467, originating from the spindle and kinetochore associated complex subunit 3 (SKA3) gene, assumes a pivotal role in the development of CRC [29]. Zou et al. investigated the specific function of circ_0000467 in CRC and elucidated its regulatory mechanism. Through their research, they observed an upregulation of circ_0000467 expression and DNMT3B mRNA expression, along with a downregulation of miR-651-5p expression in both CRC cell lines and tissues. They revealed that circ_0000467 inhibition resulted in a suppression of CRC cell proliferation, and invasion. Furthermore, their dual-luciferase reporter gene assays confirmed the direct interaction between miR-651-5p and circ_0000467. Their functional compensation experiments unveiled that the regulatory impact of circ_0000467 on the behavioral characteristics of CRC cells could be partially alleviated by the presence of miR-651-5p. Additionally, they identified that circ_0000467 potentially promotes the CRC cells proliferation and metastasis by directly interacting with miR-651-5p and inducing an overexpression in DNMT3B expression. In conclusion, the authors deduced that the interplay between circ_0000467, miR-651-5p, and DNMT3B plays a vital role in the pathogenesis of CRC. These findings propose a promising therapeutic target for CRC treatment [30].

###### Oral cancer

Oral cancers, including oropharyngeal cancers, rank as the sixth most prevalent form of malignancy on a global scale. The worldwide incidence of oral cancer is estimated to surpass 400,000 newly diagnosed cases annually [31]. Oral cancer is characterized by a discouraging prognosis, as evidenced by overall 5-year survival rates that can be as low as 40%. However, in cases where oral cancer is detected during the early stages (I and II), the survival rates can surpass 80%. Considering the unfavorable survival rates and compromised quality of life experienced by patients with oral squamous cell carcinoma (OSCC), there is an urgent need to delve into the molecular mechanisms underlying OSCC progression. Such exploration is essential for the identification of potential biomarkers or targeted sites that can be leveraged for effective treatment strategies [32, 33].

##### MiRNAs/DNMT3B axis in oral squamous cell carcinoma

MiR-379 is an example of a microRNA that is transcribed from the MIR379 genetic locus located on 14q32.31. It assumes a pivotal role in the suppression of cancer cell proliferation in OSCC [34]. Shiah and colleagues examine the intricate mechanisms involved in the abnormal suppression of gene expression related to RA signaling in OSCC. Their findings provided evidence that dysregulated miR-30a and miR-379 might serve as a potential mechanism for the repression of ADHFE1 and ALDH1A2 genes in OSCC. This repression is achieved through the targeting of DNMT3B by these microRNAs. They observed that the introduction of miR-30a and miR-379 resulted in the restoration of ADHFE1 and ALDH1A2 genes, which had been silenced due to methylation. This re-expression caused growth inhibition in oral cancer cells. Additionally, it was discovered that the perturbation of miRNAs and DNMT3B may arise as a consequence of being subjected to tobacco smoking and betel quid chewing. Their findings provide evidence that the act of betel quid chewing and tobacco smoking has the capacity to inhibit the expression of miR-30a and miR-379, molecular entities responsible for the upregulation of DNMT3B. As a result, this dysregulation prompts ADHFE1 and ALDH1A genes hypermethylation, thereby facilitating the progression of oncogenic processes. The outcomes of their investigation emphasize the prospective application of retinoids, in conjunction with epigenetic modifiers, as a viable approach for the prevention or treatment of oral cancer [35].

##### MiRNAs/DNMT3B axis in tongue squamous cell carcinoma

The miR-29 family fulfills a significant role in numerous physiological and pathological processes by modulating target genes involved in diverse biological phenomena including angiogenesis, apoptosis, survival, and proliferation [36]. Jia et al. examined expression patterns of miR-195 and miR-29b in relation to each other in cases of tongue squamous cell carcinoma (TSCC). Their findings revealed a marked downregulation of miR-195 and miR-29b in TSCC samples when compared to their corresponding nonmalignant tissues, as evidenced by a sample size of 60 paired specimens. Their investigation revealed a positive correlation between the expression levels of miR-29b and miR-195 in both TSCC samples and their corresponding nonmalignant tissues. They observed that the upregulation of miR-29b resulted in the demethylation of CpG islands located upstream of miR-195 by specifically targeting DNMT3B. Consequently, this demethylation event led to an increase in the expression of miR-195 within TSCC cell lines. During their investigation, the researchers observed that upon silencing DNMT3B, there was a notable increase in the expression of miR-195, accompanied by a reduction in CpG island methylation upstream of the miR-195 gene. Furthermore, their observations highlighted that the sole overexpression of miR-29b significantly augmented the expression of miR-195. However, when miR-29b was co-transfected with DNMT3B, no significant alteration in miR-195 expression was noted. Taken together, their findings provided compelling evidence that miR-29b is capable of inducing an upregulation of miR-195 by directly targeting DNMT3B in cases of TSCC. As a result, miR-29b/DNMT3B/miR-195 axis holds promise as a potential avenue for exploring innovative therapeutic strategies for TSCC [37].

##### LncRNA MEG3/DNMT3B axis in oral cancer

Maternally expressed gene 3 (MEG3) is situated in the genomic region of human chromosome 14q32.3 and spans approximately 1.6 kilobase in length. Multiple investigations have provided evidence demonstrating the inhibitory effects of lncRNA MEG3 on the growth and metastasis of OSCC. Consequently, there is a promising prospect for utilizing MEG3 as a novel diagnostic biomarker and therapeutic target in the management of OSCC [38]. Jia et al. sought to examine the potential involvement of the lncRNA MEG3/miR-26a/DNMT3B axis in the pathogenesis of TSCC. Their observations revealed a significant downregulation of miR-26a and lncRNA MEG3 gene expression in TSCC samples when compared to their respective levels in matched nonmalignant tissues. Additionally, the co-occurrence of low expression levels of both miR-26a and MEG3 emerged as an independent prognostic indicator associated with unfavorable clinical outcomes among patients diagnosed with TSCC. Through their experimental assays conducted on human TSCC cell lines SCC-15 and CAL27, they demonstrated that miR-26a specifically targets the DNMT3B transcript. Furthermore, the inhibition of miR-26a resulted in MEG3 overexpression, establishing a potential mechanistic connection between the observed downregulation of miR-26a and MEG3 in TSCC tissue. They also revealed that the upregulation of either MEG3 or miR-26a in CAL27 and SCC-15 cells exerted inhibitory effects on cell cycle progression and cell proliferation. Additionally, miR-26a or MEG3 upregulation facilitated cell apoptosis. Taking into account the unfavorable prognostic implications linked to diminished levels of miR-26a and MEG3, their research outcomes suggest a significant role for the lncRNA MEG3/miR-26a/DNMT3B axis in exerting crucial antitumor effects in the pathogenesis of TSCC [39].

##### CiRS-7-DNMT3B axis in oral cancer

In recent discoveries, a newly identified circular RNA called ciRS-7 has been recognized to function as a naturally occurring competing endogenous RNA, commonly referred to as a “super sponge,” for miR-7. This interaction entails the sequestration of miR-7 and the subsequent competitive inhibition of its activity. CiRS-7 has demonstrated upregulation in various cancer types, including OSCC. Its upregulation has been found to facilitate the malignant phenotype of cancer cells by enhancing cellular proliferation, and invasion both in vivo and in vitro [40]. In the study conducted by Zhao et al., the researchers investigated the underlying mechanism through which circular RNA ciRS-7 impacts laryngeal squamous cell carcinoma (LSCC). Their findings revealed a significant correlation between elevated ciRS-7 expressions in LSCC tissues and advanced clinical stage, as well as an exacerbation of tumor infiltration and lymph node metastasis in patients diagnosed with LSCC. Zhao et al. delve into the underlying mechanisms through which circular RNA ciRS-7 exerts its influence on the pathogenesis of LSCC. Within their investigation, heightened levels of ciRS-7 expression were observed within LSCC tissue samples, displaying a significant correlation with advanced clinical stages, as well as intensified infiltration and lymph node metastasis among individuals diagnosed with LSCC. The researchers revealed that the suppression of ciRS-7 resulted in diminished viability and EMT while promoting apoptosis in LSCC cells. Subsequent experimental analyses unveiled that the growth and EMT of LSCC cells were stimulated despite the silencing of ciRS-7, when either miR-432-5p was inhibited or DNMT3B was upregulated. The researchers ultimately disclosed that ciRS-7 suppression resulted in the inhibition of xenograft tumor proliferation within an in vivo setting. Therefore, ciRS-7 facilitates the advancement of LSCC by enhancing TGM3 methylation through the pivotal involvement of miR-432–5p/DNMT3B axis [41].

#### Lung cancer

Lung cancer stands as the predominant contributor to cancer-related mortality on a global scale. Notably, its prevalence is swiftly escalating in emerging nations, wherein non-small-cell lung cancer (NSCLC) encompasses over 80% of all reported instances of lung cancer [42]. Despite notable progress in the diagnosis and chemotherapeutic approaches employed to combat NSCLC, the prognosis for this malignancy continues to be poor. Regrettably, the 5-year overall survival rate for individuals afflicted with NSCLC stands at a discouragingly low 11%. Therefore, there is an immediate imperative to unravel the intricate molecular mechanisms governing the metastatic progression of NSCLC [43].

##### MiRNAs/DNMT3B axis in lung cancer

miR-152, belonging to the miR-148/152 family, exhibits a substantial reduction in expression within NSCLC cases. Notably, the augmentation of miR-152 levels exerts a significant inhibitory effect on the motility and proliferation of NSCLC cells [44]. Yang et al. investigated the epigenetic mechanism through which miR-152-3p modulates proliferation in A549 cells of NSCLC by targeting neural cell adhesion molecule 1 (NCAM1). They presented findings that revealed a comparatively elevated level of methylation within the promoter region of miR-152-3p. They successfully identified the interaction between DNMT3B protein and miR-152-3p. This binding event was observed to predominantly transpire within the central region of the miR-152-3p gene in A549 cells. By means of a luciferase assay, they determined NCAM1 as the specific gene targeted by miR-152-3p. Their experimental investigation further substantiated that miR-152-3p exhibited the capacity to suppress cell proliferation and invasion while concurrently downregulating NCAM1 expression level. The researchers observed that NCAM1 upregulation could mitigate the impact exerted by miR-152-3p. Furthermore, they revealed that the depletion of DNMT3B led to a reduction in the proliferative capacity of A549 cells along with an elevation in miR-152-3p expression, whereas it simultaneously diminished the expression of NCAM1. Conversely, miR-152-3p inhibitor administration counteracted these effects, thereby promoting an upregulation of NCAM1 expression. Their findings additionally demonstrated that miR-152-3p potentially inhibits the growth of A549 cells by decreasing NCAM1. In this manner, DNMT3B exerts a negative regulatory effect on miR-152-3p expression by modulating the methylation level in the core region of miR-152-3p. This mechanism ultimately mediates lung tumor cells proliferation [45]. Furthermore, Yang et al. conducted an inquiry to examine the potential impact of miR-203a-3p on migration, and cell proliferation across diverse cancer cell lines. In their investigation, it was observed that miR-203a-3p exhibited a downregulation pattern in both NSCLC cell lines and tissue samples, and miR-203a-3p upregulation in vitro exerts inhibitory effects on the proliferation, and invasion of NSCLC cells. Their subsequent investigations revealed that miR-203a-3p directly target DNMT3B. Moreover, they unveiled that promoter hypermethylation represents a potential mechanism underlying the diminished expression of miR-203a-3p in NSCLC. Notably, they observed a reciprocal regulation mechanism between DNMT3B and miR-203a-3p. Subsequently, they provided conclusive evidence that the miR-203a-3p upregulation leads to a reduction in tumor growth in an in vivo setting. Thereby, miR-203a-3p-DNMT3B feedback loop plays a prominent role in facilitating the progression of NSCLC [46].

##### LncRNA MEG3/DNMT3B axis in lung cancer

Recognized as a prominent regulator, the lncRNA MEG3 has been documented to function as a vital regulatory element in the context of lung cancer. Furthermore, prior investigations have provided evidence indicating a substantial reduction in the expression of lncRNA MEG3 in NSCLC. Additionally, it has been observed that diminished levels of lncRNA MEG3 expression correlate with an unfavorable prognosis among individuals diagnosed with lung cancer [47]. As well, Zhou and colleagues made a noteworthy finding that exposure to the environmental carcinogen nickel induced a downregulation of MEG3. This subsequent event initiated the inhibitory effect on c-Jun-mediated PHLPP1 transcription and led to an upregulation of hypoxia-inducible factor-1α (HIF-1α) protein translation. Consequently, these molecular alterations contributed to the malignant transformation of human bronchial epithelial cells. Their mechanistic investigation revealed that downregulation of MEG3 could be attributed to the promoter hypermethylation induced by nickel exposure, which led to an increase in DNMT3b expression. Furthermore, they observed that the transcriptional inhibition of PHLPP1 was a consequence of the diminished interaction between MEG3 and its inhibitory transcription factor, c-Jun. They revealed that the suppression of PHLPP1 led to an enhancement of HIF-1α protein translation through the activation of the Akt/p70S6K/S6 axis in response to nickel stimuli. In conclusion, their findings unveil that exposure to nickel triggers an induction of DNMT3B and a hypermethylation of the MEG3 promoter, resulting in the inhibition of MEG3 expression. This, in turn, diminishes its binding to c-Jun, leading to increased c-Jun-mediated inhibition of PHLPP1 transcription. Consequently, the activation of the Akt/p70S6K/S6 axis occurs, facilitating HIF-1α protein translation, and ultimately contributing to the malignant transformation of human bronchial epithelial cells. Their investigations offer valuable insights into comprehending the modification and function of MEG3/DNMT3B in the process of nickel-induced lung tumorigenesis [48].

#### Glioblastoma

Glioblastoma multiform (GBM) is a prevalent malignancy worldwide, characterized by a high incidence across diverse populations. Despite extensive global endeavors, GBM continues to represent a formidable illness, exhibiting a poor prognosis. Hence, there is an urgent need to elucidate the precise molecular mechanisms underlying its pathogenesis and to investigate innovative therapeutic approaches for the management of this devastating ailment.

##### MiRNAs/DNMT3B axis in glioblastoma

Recent comprehensive research has revealed that the miR-29 family assumes a pivotal role in the pathogenesis of glioma, and holds promising potential as a biomarker for glioma screening purposes [49]. Xu et al. conducted an inquiry into the involvement of miR-29 s in glioblastoma, wherein they provided evidence supporting the effective suppression of DNMT3B expression through mRNA degradation mediated by miR-29s in the U87MG glioblastoma cell line. Their findings revealed that the introduction of exogenous miR-29s significantly impeded the proliferation, and migration, capabilities of U87MG cells, while concurrently stimulating their apoptotic processes. They observed that these effects could be effectively replicated by utilizing small interfering RNA targeting DNMT3B, and were partially attenuated upon co-transfection of DNMT3B expression plasmids. This suggests that miR-29s exert their tumor-suppressive effects, at least in part, through the inhibition of DNMT3B expression. These findings offer a compelling basis for the development and implementation of therapeutic strategies targeting miR-29s/DNMT3B as a potential approach for treating glioblastoma [50].

##### circRNA_104948/DNMT3B axis in glioblastoma

Zhang et al. conducted an investigation into the role of circRNA_104948 in glioma and its underlying regulatory mechanism. Their experimental data indicated the upregulation of circRNA_104948 in plasma exosomes, tissue samples from glioma patients, and various glioma cell lines. In their study, the researchers observed that treatment of normal astrocytes with exosomal circRNA_104948 resulted in an augmentation of cell proliferation and a reduction in apoptosis. However, these effects were reversed when sh-circRNA_104948 was employed. Furthermore, the researchers identified miR-29b-3p as a novel target of circRNA_104948, and proposed DNMT3B as a potential downstream molecule regulated by miR-29b. Their subsequent experimental investigations demonstrated that circRNA_104948 had the ability to modulate the proliferation and apoptosis of astrocytes through the miR-29b-3p/DNMT3B/MTSS1 signaling pathway. Moreover, the alterations in biological behavior induced by glioma-derived exosomes were effectively reversed upon the introduction of miR-29b-3p mimics. Similarly, the increased cell growth caused by miR-29b-3p inhibitors was counteracted by the DNMT3B knockdown. Lastly, the effects mediated by miR-29b-3p mimics were nullified by the DNMT3B overexpression. Their discoveries uncovered the significant contributions of circRNA_104948 in the progression of glioma, highlighting that the circRNA_104948/miR-29b-3p/MTSS1/DNMT3B pathway holds promise as a potential therapeutic target for individuals diagnosed with glioma [51].

#### Hepatocarcinoma

Hepatocellular carcinoma (HCC) ranks as the third most common cause of cancer-related mortality globally [52]. While advancements in HCC treatment have been made, the prognosis for patients with this disease continues to be unfavorable due to a considerable recurrence rate. Enhancing our comprehension of the pathogenesis underlying the development of HCC holds significant potential in advancing diagnostic and therapeutic strategies, particularly for the identification and management of HCC during its early stages, leading to improved clinical outcomes [53].

##### MiRNAs/DNMT3B axis in hepatocarcinoma

A growing body of evidence suggests that dysregulation of miR-29c-3p expression is frequently observed in various tumor types, with a particular emphasis on its role in promoting tumor development, notably in HCC [54]. Wu et al. delved into the regulatory role of miR-29c-3p and DNA methylation in HCC. Their findings unveiled a substantial downregulation of miR-29c-3p expression in both HCC cell lines and tissues. Notably, they observed that low levels of miR-29c-3p expression correlated with larger tumor size, multiplicity of tumors, distinct pathologic features, and shorter overall survival rates in HCC patients. They revealed that the miR-29c-3p upregulation exerted a substantial inhibitory effect on HCC migration, apoptosis, and cell proliferation, and in vivo tumor growth. They discovered that DNMT3B exhibited upregulation in HCC tissues, and there existed a negative correlation between its expression and miR-29c-3p levels. They further demonstrated that DNMT3B serves as a direct target gene under the regulatory control of miR-29c-3p. Their subsequent experimentation revealed that miR-29c-3p effectively modulates the methylation status of the large tumor suppressor gene 1 (LATS1) through its interaction with DNMT3B. Importantly, they observed that aberrant methylation of LATS1 leads to the inactivation of the Hippo signaling pathway. Subsequently, they identified a high prevalence of elevated DNMT3B expression and reduced LATS1 expression in HCC tissues. Furthermore, they established that these molecular alterations were closely linked to an unfavorable prognosis in HCC patients. In conclusion, miR-29c-3p functions as a tumor suppressor in HCC by directly targeting DNMT3B and modulating the LATS1-associated Hippo signaling pathway. This novel insight suggests that targeting this regulatory axis could present a promising therapeutic strategy for the treatment of HCC [55].

##### LncRNA MEG3/DNMT3B axis in hepatocarcinoma

Recent investigations have unveiled that lncRNA MEG3 assumes a crucial role in the pathogenesis of liver cancer, highlighting its potential as a viable therapeutic target for the management of this malignancy [56]. Li et al. explored the involvement of the miR-26a/DNMT3B/MEG3 axis in HCC progression. Their findings indicate a frequent downregulation of both miR-26a and the lncRNA MEG3 in HCC tissues when compared to corresponding non-malignant tissues. The researchers firstly observed a negative correlation between the levels of miR-26a and MEG3 expression and both the sizes of tumors and the TNM clinical stage in patients diagnosed with HCC. They revealed that the miR-26a upregulation resulted in a substantial decrease in the proliferative, invasive, and migratory capabilities of HCC cells. Moreover, the researchers provided evidence indicating that the DNMT3B gene was a direct target of miR-26a. Furthermore, it was observed by the researchers that the restraining of DNMT3B expression exhibited comparable effects in suppressing tumor growth, as seen with the induction of miR-26a overexpression. This inhibition also led to the increased expression of MEG3. Subsequently, it was discovered that DNMT3B expression levels exhibited an elevation in HCC tissues when compared to non-malignant tissues. Notably, a significant inverse correlation was observed between DNMT3B and both miR-26a and MEG3 within the context of HCC tissues. In this manner, miR-26a/DNMT3B/MEG3 axis engaged in the progression of HCC [57].

#### Hematological cancer

Hematological malignancies encompass a diverse group of cancers characterized by varying incidence rates, prognosis, and underlying causes. Given their inherently aggressive nature, it is imperative to facilitate early diagnosis in order to enhance prognosis, treatment efficacy, and overall survival rates. Considering the constraints posed by existing biomarkers concerning their sensitivity, specificity, and predictive capabilities, there is a pressing need to advance the development of novel diagnostic tools and biomarkers. This undertaking aims to enable early detection and diagnosis of hematological malignancies with improved accuracy and reliability [58].

##### MiRNAs/DNMT3B axis in hematological cancer

MiR-375, an evolutionarily conserved noncoding RNA, has been implicated in critical cellular processes such as tumor cell proliferation, migration, and drug resistance. A growing body of evidence substantiates the frequent downregulation of miR-375 across diverse cancer types. Furthermore, miR-375 functions as a tumor suppressor by impeding malignant characteristics manifested by cancer cells [59]. Bi et al. explored the anti-leukemia effects exerted by miR-375 in acute myeloid leukemia (AML). Within their investigation, they observed a marked reduction in the expression levels of miR-375 in both leukemic cell lines and primary AML blasts when compared to normal control samples. This decrease was attributed to the presence of DNA hypermethylation specifically within the promoter region of precursor-miR-375 (pre-miR-375) in leukemic cells, while normal controls did not exhibit such alterations. They revealed that miR-375 induction exhibited notable effects, including the suppression of proliferation and colony formation in leukemic cells. Through their mechanistic investigation, the researchers unveiled that miR-375 elevation led to a decrease in HOXB3 expression. Additionally, they observed that miR-375 exerted its suppressive effects by binding to the 3′-UTR region of HOXB3 mRNA. They further identified that HOXB3 upregulation exhibited a partial inhibitory effect on the miR-375-induced proliferation arrest and reduction in colony numbers. This observation strongly suggests that HOXB3 plays a significant role in mediating the anti-leukemia activity induced by miR-375. Their subsequent experimental investigations conducted by the researchers unveiled that the depletion of HOXB3 using short hairpin RNAs resulted in a reduction in the expression levels of cell division cycle associated 3 (CDCA3). This downregulation of CDCA3, in turn, led to a decrease in cell proliferation. The researchers ultimately established the causative relationship between HOXB3 and DNMT3B upregulation, whereby the former stimulated the latter’s expression and facilitated its binding to the pre-miR-375 promoter. This interaction resulted in an increased level of DNA hypermethylation within the pre-miR-375 region, consequently resulting in diminished expression levels of miR-375. In this manner, the researchers successfully identified a regulatory circuitry involving miR-375, HOXB3, and CDCA3/DNMT3B plays a significant role in the development of leukemia and also presents a potential therapeutic approach for AML by targeting the restoration of miR-375 expression [60].

##### LncRNA HOTAIRM1/DNMT3B axis in hematological cancer

The divergent lncRNA HOTAIRM1 (HOXA transcript antisense RNA myeloid-specific 1) is situated within the HOXA gene cluster, precisely positioned between HOXA1 and HOXA2. It spans a length of 5415 base pairs and is located at the human chromosome 7p15.2 region. Notably, the transcription initiation site of HOXA1 shares an identical CpG island with HOTAIRM1. Initially identified for its crucial role in granulocytic differentiation within NB4 promyelocytic leukemia, HOTAIRM1 has emerged as a notable lncRNA associated with cancer. Aberrant expression of HOTAIRM1 has been observed across various tumor types, signifying its relevance in cancer-related processes [61]. Zhang and colleagues investigated the impact of benzene or hydroquinone (HQ) on the expression of HOTAIRM1 in the AML-associated pathway. Their research revealed distinct patterns of HOTAIRM1 expression in HQ-ST cells, where it initially increased before subsequently decreasing, whereas in HQ-MT cells, there was a definitive decrease. Interestingly, the observed changes in DNMT3B expression exhibited an opposite trend when compared to HOTAIRM1. Additionally, they revealed that the administration of 5-AzaC, which is a DNA methyltransferase inhibitor, or TSA, a histone deacetylation inhibitor, to HQ-MT cells resulted in the restoration of HOTAIRM1 expression. This restoration was attributed to decreased levels of DNA promoter methylation. Their subsequent functional experimentation revealed that the manipulation of HQ-MT cells through CRISPR/Cas9-mediated DNMT3B knockout led to promoter hypomethylation and a subsequent increase in HOTAIRM1 expression. Thereby, prolonged exposure to HQ or benzene could potentially result in an upregulation of DNMT3B expression and subsequent promoter hypermethylation. This hypermethylation effectively silences HOTAIRM1 expression, which is believed to be a tumor suppressor involved in the AML-associated carcinogenesis pathway [62].

#### Breast cancer

Breast cancer, a prevalent malignancy affecting women, is characterized by its heterogeneous nature and intricate pathogenesis. Despite extensive research, the precise mechanisms underlying its progression remain inadequately elucidated. Therefore, there is a need for enhanced understanding of the intricate molecular mechanisms that drive disease progression in breast cancer. Furthermore, the development of effective targeted therapies is imperative to meet the specific treatment requirements of patients afflicted with this condition.

##### MiRNAs/DNMT3B axis in breast cancer

Emerging experimental findings indicate that miR-29c exerts a noteworthy influence in impeding the advancement of breast malignancies, thereby signifying its potential utility as a discriminative indicator for breast cancer diagnostics [63]. Li et al. comprehensively examined the role and underlying mechanism of miR-29c in breast cancer. They initially observed a notable downregulation of miR-29c expression in breast cancer cells, and demonstrated that an elevated expression of miR-29c resulted in the suppression of cellular proliferation, invasion, colony formation, and growth within a 3D Matrigel environment. Additionally, it was observed that miR-29c exhibited a binding affinity towards the 3′UTR of DNMT3B and exerted a suppressive effect on its expression. Subsequent experimental investigations unveiled that the downregulation of miR-29c in cells resulted in a decrease in the protein expression of TIMP3, accompanied by an increase in the methylation levels of the TIMP3 gene when compared to control cells. Conversely, the upregulation of miR-29c led to an elevation in TIMP3 protein levels and a reduction in TIMP3 methylation levels relative to control cells. Conclusively, DNMT3B suppression through knockdown techniques resulted in decreased cellular proliferation, and invasion within breast cancer cell lines. Based on these observations, miR-29c exerted its functional role in breast cancers by modulating the DNMT3B/TIMP3/STAT1/FOXO1 pathway [63]. Furthermore, Tang et al. explored the molecular mechanism responsible for maintaining the active state of cancer-associated fibroblasts (CAFs). Their findings revealed that in CAFs, DNMT3B served as a target for miR-200b, miR-200c, and miR-221, while also being capable of inducing DNA methylation of the promoters of miR-200s. In the context of prolonged exogenous TGF-β1 treatment in normal fibroblasts (NFs), DNMT3B gradually attained a sustained elevation due to a dual influence: the diminishing levels of miR-200b/c and the escalating levels of miR-221. Consequently, DNMT3B played a pivotal role in modulating CAF activation by exerting a suppressive effect on the expression of miR-200s, leading to their reduced abundance. They revealed that the miR-200s/miR-221/DNMT3B axis played a crucial role in supporting autocrine TGF-β1 signaling, which in turn maintained the active status of CAFs. Additionally, disruption of the autocrine TGF-β1/miR-200s/miR-221/DNMT3B axis resulted in the miR-200s promoter's demethylation and consequent restoration of the NF phenotypes. They additionally validated that TCF12, the specific gene regulated by miR-141, provokes the c-Myc/Cyclin D1 pathway within breast cancer cells, thus promoting cancerous proliferation through augmentation of CXCL12 expression in CAFs. In this manner, the regulatory loop involving TGF-β1/miR-200s/miR-221/DNMT3B plays a crucial role in sustaining the phenotypic characteristics of CAFs and is indispensable for their functional involvement in facilitating the progression of breast cancer. This intricate regulatory network presents a promising target for therapeutic interventions aimed at inhibiting CAF-driven malignancy [64].

##### LncRNA RAMP2-AS1/DNMT3B axis in breast cancer

RAMP2-AS1, an exemplar lncRNA originating from the opposite strand of RAMP2, serves as a plausible gene in the divergence of aging patterns in endothelial cells (ECs). Contemporary scrutiny has exposed the capacity of RAMP2-AS1 to serve as an innovative biomarker and therapeutic target for various human malignancies [65]. Li et al. aimed to explore the potential involvement of DNMT3B/RAMP2-AS1 in the pathogenesis of breast cancer. Their investigation revealed a notable downregulation in the expression level of RAMP2-AS1 in both breast cancer tissues and cells, while CXCL11 exhibited significantly elevated expression levels. They unveiled a significant association between RAMP2-AS1 downregulation and unfavorable prognostic outcomes in patients. Moreover, RAMP2-AS1 exerted inhibitory effects on the malignant characteristics of breast cancer cells. Furthermore, they revealed that RAMP2-AS1 played a regulatory role in the methylation process of CXCL11 by facilitating the recruitment of DNMT1 and DNMT3B to the promoter region of CXCL11. In conclusion, RAMP2-AS1 exerts a suppressive effect on the malignant characteristics of breast cancer through the inhibition of CXCL11, mediated by DNMT1 and DNMT3B [66].

#### Bladder cancer

Bladder cancer (BCa) ranks as the fifth most prevalent form of cancer globally and exhibits substantial implications for both individual well-being and mortality rates. The clinical outlook for muscle invasive BC is unfavorable, with high rates of recurrence following radical surgery or chemotherapy. Consequently, the development of novel diagnostic techniques and treatment approaches assumes paramount importance in addressing this pressing issue [67].

##### MiRNAs/DNMT3B axis in bladder cancer

A growing body of evidence suggests that miRNAs contribute to bladder cancer development, progression and metastasis [68]. Zo et al. investigated the impact of miR-124-3p on BC through modulation of DNMT3B. Their investigation revealed a noteworthy downregulation of miR-124-3p expression and a concomitant upregulation of DNMT3B levels in both BCa tissues and cell lines, as compared to corresponding normal controls. They confirmed the direct targeting of DNMT3B by miR-124-3p. Additionally, they revealed that the introduction of miR-124-3p mimics and DNMT3B siRNAs resulted in decreased proliferation, and invasion of BCa cells, along with increased cell apoptosis. Conversely, the inhibition of miR-124-3p led to enhanced proliferation, migration, and decreased apoptosis of breast cancer cells. Furthermore, they observed that the impact of DNMT3B cDNAs could be attenuated when combined with miR-124-3p mimics. Thus, action of miR-124-3p on BC cells involves the repression of proliferation, and invasion, as well as the facilitation of apoptosis. These effects are attributed to the direct targeting of DNMT3B by miR-124-3p [69]. Furthermore, Xu et al. explored the potential involvement of DNMT3B in BCa through miR-34a promoter methylation. They firstly revealed that the expression of miR-34a was significantly elevated in BCa tissues exhibiting low levels of DNMT3B expression, in contrast to BCa tissues characterized by high levels of DNMT3B expression. Their discovery revealed a positive correlation between the methylation ratio of the miR-34a promoter and the level of DNMT3B. They unveiled that the suppression of DNMT3B resulted in an augmentation of miR-34a expression and enhanced transcriptional activity of the miR-34a promoter, along with a reduction in miR-34a promoter methylation. Notably, inhibition of DNMT3B resulted in suppressed migration and invasion capabilities, accompanied by a decline in the protein levels of hepatocyte nuclear factor 4 gamma (HNF4γ) and Notch1. These proteins are recognized as downstream targets influenced by miR-34a. Ultimately, they discovered that the observed inhibitory effects of DNMT3B were alleviated when using a miR-34a inhibitor. As a result, suppression of DNMT3B hampers migration in BC by facilitating the epigenetic promotion of miR-34a [70]. Furthermore, Ying et al. provided insights into the functional significance of miR-502-5p within the context of BCa. Their findings revealed consistent downregulation of miR-502-5p in BCa, with the underlying mechanism involving hypermethylation of CpG islands as a contributing factor for such downregulation. Their functional experiments revealed the inhibitory effects of miR-502-5p upregulation on cellular proliferation and migration in vitro, as well as its suppressive impact on tumor growth in vivo. Furthermore, they successfully identified DNMT3B as a direct target of miR-502-5p. In this manner, miR-502-5p/DNMT3B regulatory network holds promise as a potential avenue for the advancement of more efficacious therapeutic approaches aimed at combating BCa [71]. In addition, Liu et al. explored the functional significance of miR-451a in the context of BCa, wherein DNMT3B played a contributory role. The authors observed a notable reduction in the expression levels of miR-451a within both the serum samples of BCa patients and cultured cell lines. They identified a notable elevation in the expression of DNMT3B within BCa cells. Consequently, this led to the facilitation of methylation processes targeting the miR-451a promoter, ultimately resulting in the repression of miR-451a. Furthermore, they indicated that miR-451a specifically targeted and exerted a negative regulatory effect on the Erythropoietin-producing hepatocellular receptor tyrosine kinase class A2 (EPHA2) gene. Conversely, it was found that EPHA2 had the capacity to activate the PI3K/AKT signaling pathway, thereby stimulating the growth and metastasis of BCa cells. In this manner, downregulation of miR-451a, facilitated by DNMT3B, plays a crucial role in promoting the proliferation, and invasion of BCa cells. This finding holds promise for the development of more efficacious therapeutic strategies targeting BCa [72].

##### LncRNA H19/DNMT3B axis in bladder cancer

LncRNA H19, which was among the earliest identified lncRNAs, is transcribed from the H19 gene situated in the chromosomal region 11p15.5. H19 has been observed to exhibit aberrant expression in various tumor types. In these contexts, it promotes several oncogenic phenotypes, including modulation of glucose metabolism, colony formation, enhanced cellular viability, cell cycle progression, EMT, motility, growth, and autophagy [73]. Lv et al. examined the involvement of LncRNA H19 in the development of tumorigenesis in BCa. Their findings revealed a notable co-expression of DNMT3B and H19 in both BCa tissues and cells. A higher degree of co-expression between H19 and DNMT3B was observed in both BC tissues and cells. Subsequently, they revealed that H19 had the ability to enhance proliferation, and migration of BC cells in vitro. They revealed that aberrant overexpression of H19 resulted in the stimulation of tumorigenesis, angiogenesis, and pulmonary metastasis within an organism. Conversely, H19 suppression demonstrated opposite effects both in vitro and in vivo. Their mechanistic investigation validated that H19 possesses the ability to directly interact with miR-29b-3p and relieve the repression of DNMT3B, thereby inducing its expression. Importantly, they observed that the augmentation of H19 expression hindered the proliferative, migratory, and EMT-suppressive effects mediated by miR-29b-3p in breast cancer cells. Conversely, when H19 was suppressed, it partially restored the inhibitory impact of miR-29b-3p on DNMT3B and facilitated the miR-29b-3p-induced EMT process. Taken together, H19 act as a competing endogenous RNA (ceRNA) for miR-29b-3p, thereby alleviating its inhibitory effects on DNMT3B. This interplay resulted in the promotion of EMT and subsequent metastasis in BCa. In this manner, H19/miR-29b-3p/DNMT3B axis function as a prospective target for therapeutic interventions aimed at tackling BCa [74].

#### Pancreatic cancer

Pancreatic cancer (PC) is an aggressive malignant tumor associated with a significant fatality rate. Despite the implementation of comprehensive therapeutic approaches, the long-term survival rate for PC remains exceedingly poor. Owing to its inconspicuous anatomical location, PC often evades detection until advanced stages, characterized by severe clinical manifestations. Consequently, the early identification and diagnosis of PC pose substantial clinical hurdles. Furthermore, despite the implementation of aggressive treatment, the therapeutic effectiveness of PC remains inadequate, necessitating the urgent development of novel diagnostic techniques and treatment approaches [75].

##### MiRNAs/DNMT3B axis in pancreatic cancer

The involvement of miR-29 in the process of stromal deposition is of utmost importance, as it actively suppresses the pro-growth impacts exerted by pancreatic stellate cells (PSCs) on the formation of pancreatic cancer colonies [76]. Wang et al. examined the connections between miR-29 and DNMT3B in relation to pancreatic cancer. Their analysis revealed a noticeable reduction in the expression levels of miR-29b, along with an upregulated mRNA expression of DNMT3b, within the pancreatic cancer tissues when compared to the corresponding paracancerous tissues. In their study, augmentation in protein expression of DNMT3B were detected. In addition, a significant inverse correlation was observed between DNMT3B and miR-29b in pancreatic cancer tissues. This relationship was further substantiated through luciferase reporter assays, which demonstrated the direct targeting ability of miR-29b on DNMT3B in an in vitro setting. Notably, they revealed that the upregulation of miR-29b had the capacity to reduce cell viability and induce apoptosis by directly targeting DNMT3B. Consistent outcomes were observed both in vitro and in vivo when DNMT3B was knocked down, further supporting these findings. They indicated that miR-29b could serve as a tumor suppressor in pancreatic cancer, highlighting its potential application as a therapeutic agent by targeting DNMT3B [77]. Additionally, Wang et al. revealed that miR-29b exhibited a suppressive effect on major cellular processes including migration and cellular proliferation. Additionally, it was observed that the depletion of SOX12 and DNMT3B successfully impeded the malignant activity of SW1990 cells. Their experimentation provides clear support for the direct modulation of SOX12 and DNMT3B by miR-29b via precise interaction with the 3'-UTRs of these genes. They further revealed that the suppressive effects of miR-29b on cell proliferation, and invasion were effectively counteracted by the SOX12 upregulation, along with restoration of DNMT3B expression levels. In this manner, miR-29b exerted inhibitory effects on the processes of proliferation, and invasion in pancreatic cancer cells. This inhibition was achieved through the direct targeting of two specific genes, namely SOX12 and DNMT3B [78].

#### Cholangiocarcinoma

Cholangiocarcinoma (CCA) refers to a type of malignant tumor originating from the epithelial cells, specifically found in the area extending from the intrahepatic bile ducts to the distal portion of the common bile duct where it meets the ampulla of Vater. The efficacy of existing chemotherapy regimens for cholangiocarcinoma is constrained, leading to unfavorable prognoses for patients with this condition. Hence, there is a pressing need for innovative strategies and methodologies in the treatment of cholangiocarcinoma [79].

##### MiRNAs/DNMT3B axis in cholangiocarcinoma

Cao and colleagues conducted an inquiry into the potential association between aberrant expression of miR-29b and cholangiocarcinoma. They firstly revealed that the heightened expression of miR-29b in QBC939 cells hindered cellular proliferation, triggered arrest at the G1 phase of the cell cycle, and facilitated apoptosis. They further unveiled a reduction in methylation patterns within the promoter region of the cell cycle inhibitor gene CDKN2B in cells exhibiting heightened miR-29b expression. Moreover, they substantiated the inhibitory impact of miR-29b on DNMT3B expression by means of luciferase reporter assays. Furthermore, an inverse association between DNMT3B and miR-29b was observed in clinical specimens of cholangiocarcinoma. Their additional investigations revealed that heightened DNMT3B expression facilitated cell proliferation and suppressed apoptosis in QBC939 cells. In this manner, miR-29b by direct targeting DNMT3B operates as an inhibitory factor for tumor growth in cholangiocarcinoma by alleviating the repressive influence of DNMT3B on CDKN2B expression [80].

#### Osteosarcoma

Osteosarcoma, which predominantly affects children and adolescents, ranks as the third most prevalent form of bone cancer in this age group. When a combination therapy approach consisting of neoadjuvant chemotherapy, surgery, and adjuvant chemotherapy is employed, patients without metastatic disease upon initial diagnosis exhibit a 5-year survival rate ranging from 60 to 70%. However, for individuals who exhibit metastatic disease or experience tumor recurrence, the prognosis is markedly unfavorable, with survival rates falling below 30% and 20% correspondingly. Consequently, it becomes imperative to explore novel therapeutic targets and strategies in order to address this pressing clinical challenge [81].

##### MiRNAs/DNMT3B axis in osteosarcoma

A recent investigation demonstrated that the augmented expression of miR-29a effectively impeded cellular adhesion, and migration in HOS and MG-63 cells. These findings suggest that miR-29a holds potential as a promising therapeutic target for osteosarcoma (OS) [82]. Gong et al. examined the mechanisms involving the miR-29a/DNMT3B/ suppressor of cytokine signalling 1 (SOCS1) axis and its influence on the invasion and migration processes in OS. They elucidated that DNMT3B serves as a direct target of miR-29a. They revealed that the miR-29a mimic's upregulation resulted in a decrease in DNMT3B expression and the SOCS1 methylation level. Consequently, this led to the SOCS1 overexpression in U2OS and MG-63 cells. Furthermore, they revealed that introducing miR-29a mimics through transfection facilitated apoptosis and hindered the invasion, and EMT process of OS cells. Simultaneously, it significantly decreased the translocation of p65 to the nucleus and the degradation of IκB-α. Notably, their discovery demonstrated that the co-treatment of 5-aza-2′-deoxycytidine with miR-29a mimics synergistically augmented the aforementioned effects. In conclusion, miR-29a through the direct targeting of DNMT3B and subsequently inhibition of the SOCS1/NF-κB signaling pathway facilitated apoptosis while impeding the invasion of OS cells [83].

#### Melanoma

Melanoma represents a neoplastic condition originating from melanocytes, specialized cells responsible for synthesizing melanin pigment in order to safeguard the skin against harmful ultraviolet radiation. Although early-stage melanoma exhibits a commendable survival rate of up to 90%, the prognosis for advanced metastatic melanoma is considerably grim, with a mere 10% survival rate and a notable recurrence risk reaching as high as 60%. Consequently, timely detection holds utmost significance in enhancing survival rates, necessitating immediate development of efficacious interventions for the treatment of malignant melanoma [84].

##### MiRNAs/DNMT3B axis in melanoma

**Fig. 3:**
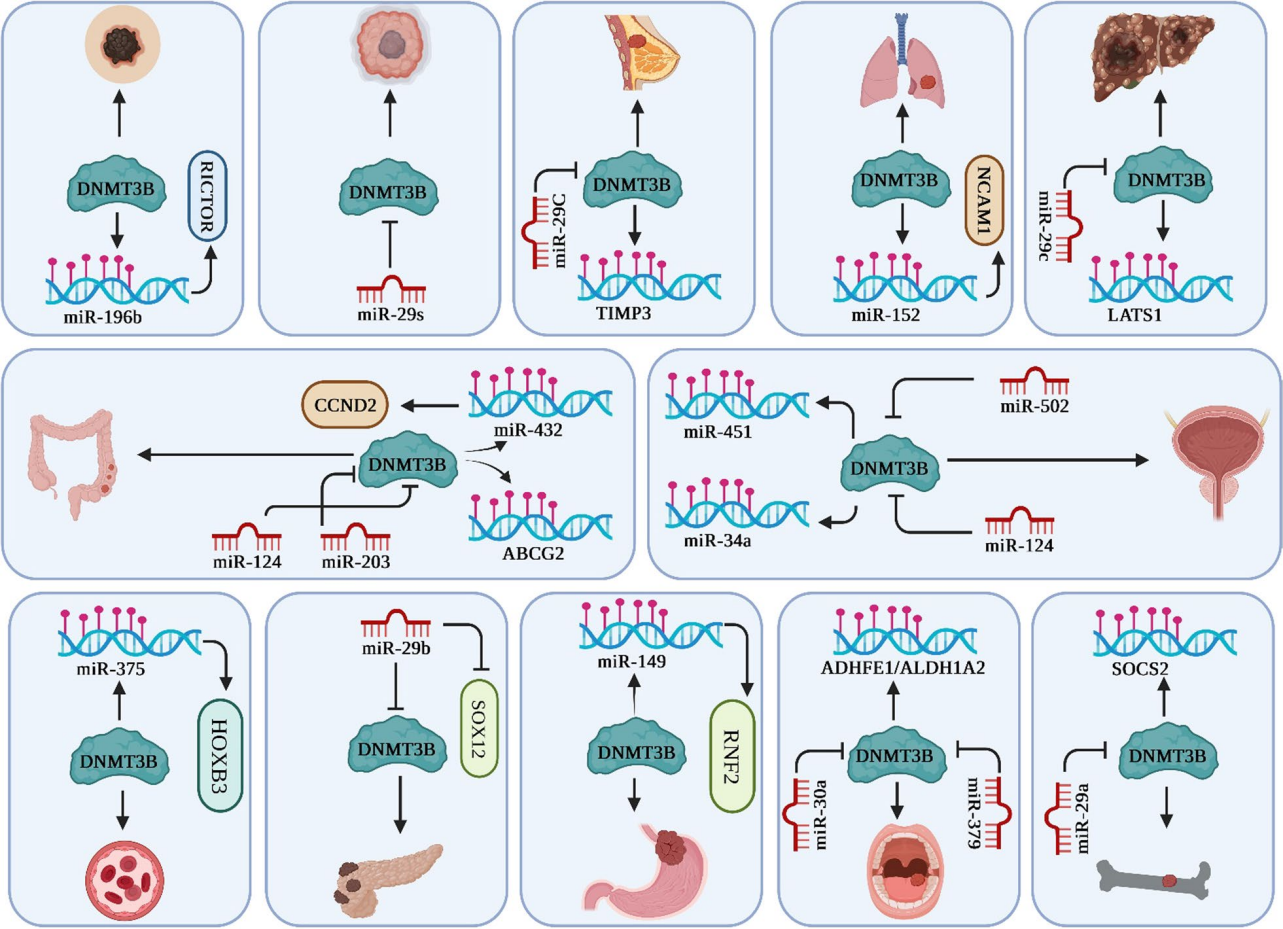
miRNAs significantly regulates DNMT3B in human cancer

**Fig. 4:**
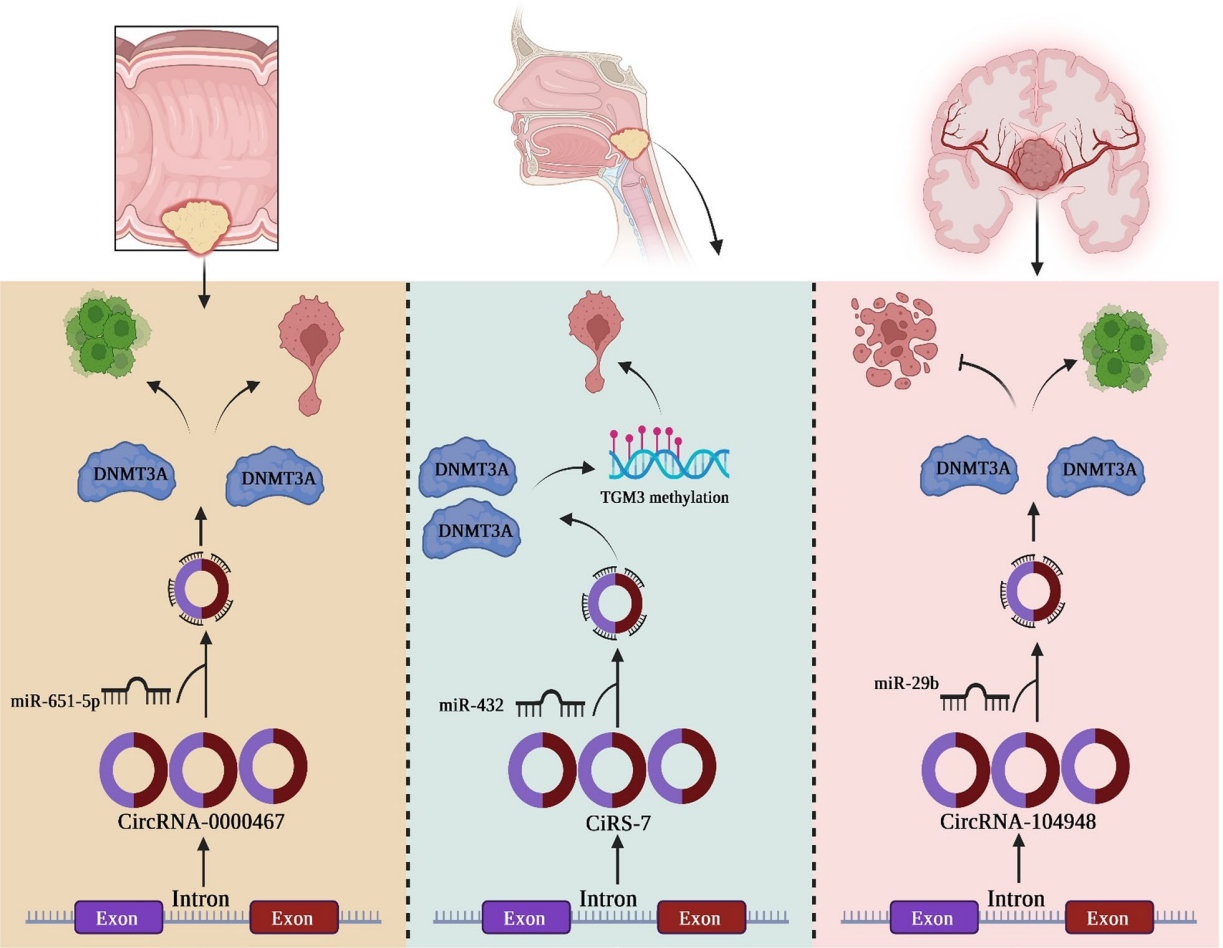
CircRNAs significantly regulates DNMT3B in human cancer

**Fig. 5:**
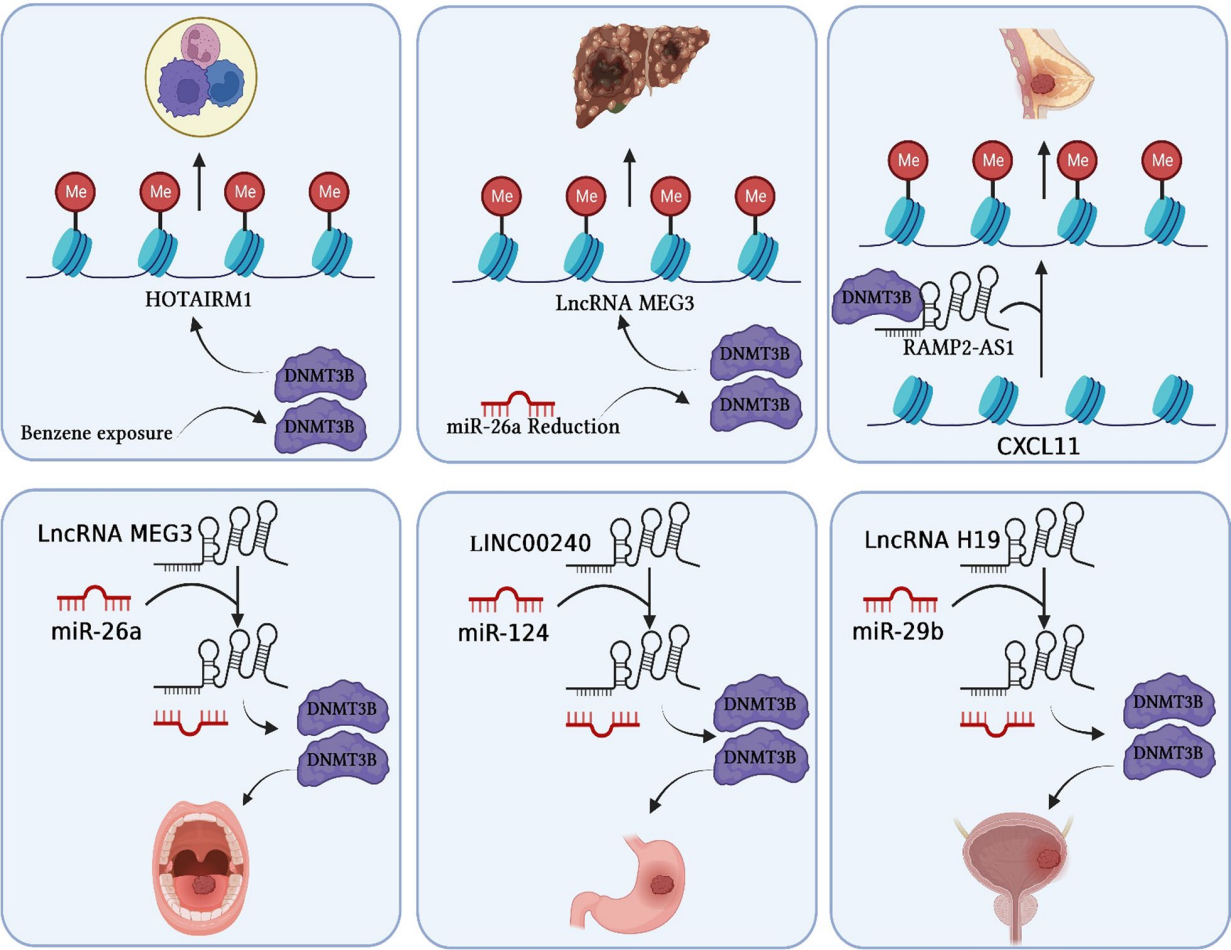
LncRNAs significantly regulates DNMT3B in human cancer

The miRNA known as miR-196s is encoded at three distinct paralogous loci within three HOX clusters, and it functions as an oncogenic miRNA in the progression of cancer [85]. Micevic and colleagues explored the precise signaling pathways regulated by DNMT3B in melanoma. Their findings indicate that DNMT3B exerts a pro-tumorigenic function in human melanoma, while its depletion significantly inhibits melanoma development in the Braf/Pten mouse melanoma model. They revealed that the DNMT3B downregulation led to a state of reduced methylation in the promoter region of miR-196b, leading to an elevation in miR-196b expression. This heightened expression consequently engages in direct modulation of Rictor, a constituent of the mTORC2 complex. They additionally observed that the RICTOR suppression hampers mTORC2 activity, a pivotal process in the initiation and progression of melanoma. These outcomes firmly establish Dnmt3B as a modulator of melanoma formation by influencing miR-196b and subsequently regulating the signaling pathways associated with mTORC2. DNMT3B/miR-196b/Rictor axis holds promise as a viable therapeutic target for the treatment of melanoma [86] (Figs. 3, 4, 5).

### Non-coding RNAs-DNMT3B in therapy resistance

In recent years, notable advancements have been made in the realm of systemic treatment options for various types of cancers. Nonetheless, a significant portion of patients eventually acquire resistance to these treatments, leading to disease progression and diminished overall survival rates. Biomarkers that can effectively indicate the escalating resistance to cancer therapies have not yet been incorporated into standard clinical practices. Nevertheless, mounting evidence substantiates the involvement of ncRNA-DNMT3B regulatory network in the initiation and advancement of cancer. Additionally, identification of their role in the emergence of drug resistance in diverse cancer types is increasingly being unveiled. As a result, there has been a proposal to explore the potential of the non-coding RNA-DNMT3B axis as influential components in innovative therapeutic approaches aimed at combating drug resistance in cancer. Consequently, the subsequent section delves into an in-depth examination of the role played by the recently identified ncRNA-DNMT3B axis in the development of drug resistance specifically within the context of cancer.

#### MiR-145/DNMT3B axis in cancer therapy resistance

Xue et al. explored the association between DNMT3B, and miR-145, as well as their response to irradiation. They observed that miR-145 exhibited a downregulatory effect on DNMT3B expression by targeting DNMT3B mRNA 3′-UTR. Moreover, they observed that DNMT3B suppression led to an increase in miR-145 expression through CpG island promoter hypomethylation. These findings indicate the presence of a significant interplay between miR-145 and DNMT3B, facilitated by a double-negative feedback loop. Their findings revealed a significant inverse relationship between the reactions of miR-145 and DNMT3B upon exposure to irradiation. Additionally, it was observed that increasing the expression of miR-145 or suppressing the activity of DNMT3B resulted in heightened sensitivity of prostate cancer cells to X-ray radiation. Their discoveries contribute to a deeper understanding of the intricate connections between miRNA and DNMTs in both cancer development and irradiation stress. Furthermore, these findings provide insights into the promising prospect of utilizing a combination of ionizing radiation and epigenetic regulation as a therapeutic approach for prostate cancer [87].

#### MiR-492/DNMT3B axis in cancer therapy resistance

Wu et al. explored the potential of down-regulating miR-492 as a means to enhance the effectiveness of cisplatin-induced cell death in gastric cancer. The researchers assess the expression levels of miR-492 in two sets of cell lines, namely SGC7901/SGC7901 CDDP resistance and AGS/AGS CDDP resistance. Their findings demonstrated that upregulation of miR-492 was observed in the cell lines exhibiting resistance to cisplatin treatment. Next, they noticed that miR-492 inhibition facilitated the efficacy of cisplatin-induced cell death in gastric cancer cells that demonstrated resistance to cisplatin. Additionally, DNMT3B upregulation through the utilization of a DNMT3B plasmid enhanced the sensitivity of gastric cancer cells with cisplatin resistance to cisplatin-induced cell death. Their findings also revealed that the DNMT3B upregulation in conjunction with cisplatin treatment significantly increased the apoptotic rate of these resistant gastric cancer cells. Importantly, they noticed that suppression of miR-492 significantly reversed the chemotherapy resistance of GC cells by inhibiting GC stemness through the targeted modulation of DNMT3B. These results provide compelling evidence that the inhibition of miR-492 represents a promising and innovative approach for managing chemoresistance in GC [88].

#### MiR-498/DNMT3B axis in cancer therapy resistance

Zhou et al. explored the involvement of miR-498 in the development of radiotherapy resistance in EC, along with its implicated mechanism. Their comprehensive analysis revealed elevated levels of DNMT3B expression and decreased levels of miR-498 expression within EC tissues. They revealed that EC tissues exhibiting radiosensitivity exhibited elevated levels of miR-498 and reduced expression of DNMT3B compared to EC tissues displaying radioresistance. Additionally, they unveiled that miR-498 upregulation or the DNMT3B suppression intensified the radiosensitivity of EC cells. They discovered that miR-498 acts as a direct regulator of DNMT3B, and the presence of DNMT3B hinders the radiosensitization process mediated by miR-498 in EC cells. In this manner, miR-498 enhances the radiation sensitivity of EC cells by inhibiting DNMT3B, thereby exerting its biological effects through the inactivation of the PI3K/AKT signaling pathway [89].

#### MiR-20a-5p/DNMT3B axis in cancer therapy resistance

Li et al. investigated the molecular mechanism underlying the role of miR-20a-5p in conferring resistance to cisplatin in ovarian cancer (OvCa). Their findings demonstrated that miR-20a-5p exhibits suppressive effects on malignant phenotypes and autophagy in OvCa cells with resistance to cisplatin. They revealed that the process of DNA methylation, specifically facilitated by DNMT3B, hinders the oncogenic and autophagic functions of RBP1 in OvCa. By conducting rescue experiments, they verified that miR-20a-5p suppresses autophagy and confers resistance to cisplatin treatment in OvCa cells by influencing DNMT3B-mediated DNA methylation of RBP1. Collectively, miR-20a-5p/DNMT3B/RBP1 axis involved in the regulation of autophagy and the development of cisplatin resistance in OvCa. These findings provide valuable insights into the potential for novel therapeutic approaches in effectively managing OvCa [90].

#### MiR-200b/c/DNMT3B axis in cancer therapy resistance

Liu et al. explored the pivotal miRNAs implicated in the modulation of drug resistance in ovarian cancer cells. Their findings revealed dysregulation of miR-200b and miR-200c in OvCa, and demonstrated that upregulation of these specific miRNAs enhanced the susceptibility of epithelial ovarian cancer (EOC) cells to cisplatin-induced cell death. Significantly, it was observed that miR-200b/c exerted a reversal effect on cisplatin resistance by targeting DNMT3A/B. They also provide compelling evidence that the regulatory influence of miR-200b/c on DNMTs plays a pivotal role in the cellular response to the administration of cisplatin. In this manner, downregulation of DNMTs through miR-200b/c signifies a potential avenue for enhancing the efficacy of chemotherapy in ovarian cancer treatment. This mechanism may heighten the sensitivity of cancer cells to chemotherapeutic agents, thereby exerting a notable influence on therapeutic outcomes [91].

#### MiR-29b/DNMT3B axis in cancer therapy resistance

Yan et al. investigated the function and underlying mechanism of miR-29b in cisplatin-resistant prostate cancer cells (PCa). Through their investigation, they observed a downregulation of miR-29b expression in PCa tissues when compared to match adjacent nontumor tissues. Additionally, they revealed that the miR-29b were significantly lower in the androgen-independent PCa cell line (LNCaP-AI) compared to the androgen-dependent PCa cell line (LNCaP). Their subsequent investigations demonstrated that overexpression of miR-29b exerted inhibitory effects on cellular proliferation and invasion while triggering apoptosis in PCa cells. Moreover, it was observed that this overexpression enhanced the susceptibility of PCa cells to cisplatin, thereby increasing their chemosensitivity. Additionally, DNMT3B and AKT3 were identified as target genes modulated by miR-29b in PCa. They found that miR-29b manifests its tumor-suppressive function by engaging in epigenetic regulation and influencing the PI3K/AKT pathway. Consequently, targeting miR-29b/DNMT3B/PI3K/AKT signaling may present a promising strategy for augmenting the therapeutic efficacy of cisplatin in the context of PCa treatment [92].

#### LncRNA HOTAIR/DNMT3B axis in cancer therapy resistance

**Fig. 6:**
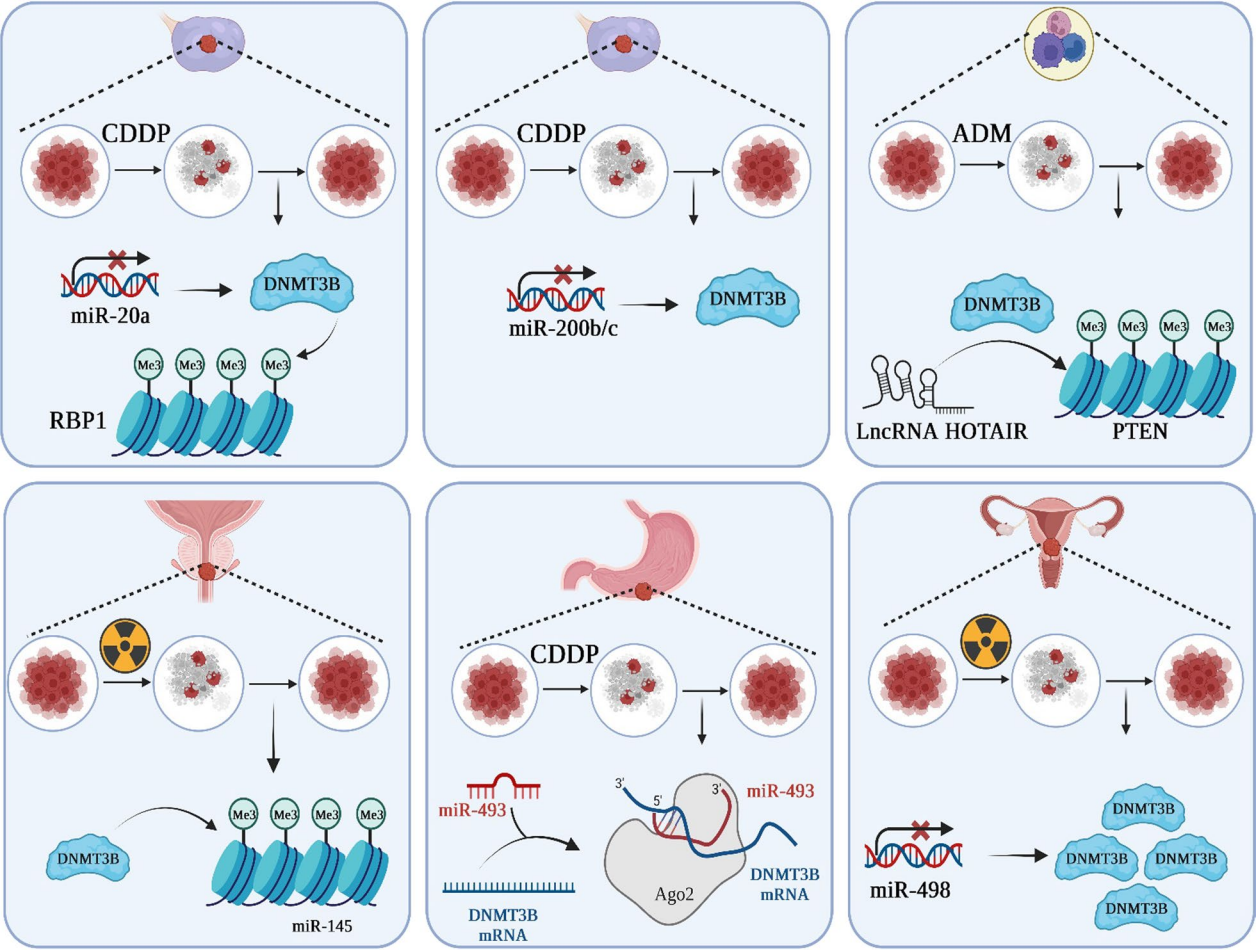
DNMT3B/ncRNAs axis in cancer therapy resistance

Zhou et al. investigated the potential involvement of HOTAIR in conferring resistance to doxorubicin (ADM) in acute myeloid leukemia (AML). Their research revealed a distinct pattern wherein patients with relapsed/refractory AML exhibited the highest levels of HOTAIR expression alongside the lowest expression of PTEN, followed by newly diagnosed AML patients and subsequently healthy controls. They observed that the administration of ADM resulted in elevated cell viability and increased IC50 values specifically in HL60/ADM cells as compared to HL60 cells. Furthermore, they unveiled a significant up-regulation of HOTAIR and concurrent down-regulation of PTEN in HL60/ADM cells. Subsequent investigations unveiled that cellular transfection involving sh-HOTAIR or pcDNA3.1-PTEN resulted in heightened sensitivity to Adriamycin (ADM), an increased rate of apoptosis, as well as reduced IC50 and cell clones. Conversely, these expression patterns could be reversed through co-transfection of pcDNA3.1-PTEN and pcDNA3.1-HOTAIR. Additionally, they noticed methylation occurring in the PTEN promoter. Notably, they demonstrate that reducing HOTAIR expression results in decreased expression of DNMT3b, with minimal impact on DNMT3a expression. Therefore, HOTAIR has the capability to stimulate PTEN methylation by increasing the levels of DNMT3b. Therefore, HOTAIR could trigger PTEN methylation by increasing the levels of DNMT3b. The researchers concluded that HOTAIR inhibits PTEN by promoting DNMT3B-dependent mechanisms and contributes to acquired drug resistance in AML [93] (Fig. 6).

### Non-coding RNA-DNMT3B axis in cancer stem cell

Cancer stem cells (CSCs) represent a minority of cancer cells with the capacity for self-renewal and differentiation. These CSCs play crucial roles in initiating tumors, facilitating tumor metastasis and recurrence, as well as contributing to the development of resistance against therapeutic drugs. Exploring the molecular mechanisms essential for the CSC population's formation has the potential to unveil innovative therapeutic approaches for treating cancer. Recent in-depth research has revealed that the ncRNA-DNMT3B axis plays a crucial role in regulating the characteristics of CSCs. In this regard, Roscigno et al. conducted a study to investigate the functional significance of miRs in human breast cancer stem cells (BCSCs), which are commonly referred to as mammospheres. Initially, it was observed that the expression of miR-221 exhibited overexpression in BCSCs when compared to their differentiated counterparts. Subsequently, it was revealed that mammospheres derived from T47D cells displayed a higher level of miR-221 in comparison to the differentiated cells. In addition, they provided additional evidence by demonstrating that the introduction of miR-221 into T47D cells through transfection resulted in an augmentation of both mammosphere formation and the expression of stem cell markers. Furthermore, through their investigations, they successfully identified DNMT3b as one of the targets of miR-221. Furthermore, in the context of BCSCs, they discovered that DNMT3b exerted a suppressive effect on the expression of several genes associated with stemness, including Oct 3/4 and Nanog. This repression was achieved through its involvement in the methylation process occurring at the promoters of these genes. Importantly, this mechanism partially counteracted the impact of miR-221 on stemness properties. In conclusion, miR-221 plays a role in promoting tumorigenicity in breast cancer by modulating stemness characteristics, primarily by exerting control over the expression of DNMT3b [94].

### Therapeutic perspective of ncRNAs-DNMT3B axis: from herbal medicine to molecular therapy

Emerging experimental evidence suggests the participation of the ncRNA/DNMT3B axis in the advancement, proliferation, and metastasis of human malignancies. Consequently, the ncRNA/DNMT3B axis holds promise as a therapeutic target for the management of human cancer. A number of inhibitors targeting the ncRNA/DNMT3B axis have been developed with the aim of treating cancer. In the subsequent section, we explore the significance of the ncRNA/DNMT3B axis as a target in various therapeutic strategies employed for the treatment of human malignancies.

#### NcRNA/DNMT3B axis modulation via herbal medicine for cancer therapy

Herbal extracts have historically been acknowledged for their antimicrobial, antidepressant, anti-inflammatory, expectorant, immune-enhancing, and potential anticancer properties. Similar to other therapeutic modalities, individuals may choose to utilize herbal medicine as a means to promote overall well-being [95]. Among the realm of alternative and complementary medicines, herbal remedies are frequently employed by individuals, particularly those who have been diagnosed with various types of cancer [96]. Fucoidan, a sulfated polysaccharide with a high fucose content, is widely distributed in marine environments and can be derived from various sources such as sea cucumbers and brown algae. It is primarily obtained from the extracellular matrix of brown algae. Fucoidan, found in diverse brown algae species, comprises l-fucose, sulfate groups, and minor constituents including xylose, mannose, galactose, rhamnose, arabinose, glucose, glucuronic acid, and acetyl groups. A broad spectrum of biological activities has been demonstrated, encompassing various aspects such as suppression of inflammation, inhibition of cancer growth, prevention of viral infections, antioxidation effects, reduction of blood coagulation and thrombosis risks, inhibition of blood vessel formation, and combat against Helicobacter pylori, among others. Previous studies disclosed that fucoidan possesses the capacity to directly exhibit anti-cancer effects by interrupting the cell cycle and inducing apoptosis, among other mechanisms. Furthermore, fucoidan can indirectly eliminate cancer cells by stimulating the activation of natural killer cells, macrophages, and similar components of the immune system. Due to its remarkable biological potency, extensive availability, minimal susceptibility to drug resistance, and limited adverse effects, fucoidan has gained prominence as a novel anti-tumor medication or adjunctive therapy when combined with existing anti-tumor drugs. The anti-tumor efficacy of fucoidan in HCC was examined by Yan et al. The researchers observed a significant overexpression of miR-29b expression in human HCC cells upon treatment with fucoidan. Furthermore, they observed a dose-dependent inhibition of DNMT3B, a downstream target of miR-29b, concomitant with the induction of miR-29b. Fucoidan exhibited a substantial reduction in luciferase activity in the DNMT3B 3′-UTR reporter, comparable to the effects observed with the miR-29b mimic. This observation strongly suggests that fucoidan induces the upregulation of miR-29b, which subsequently leads to the suppression of DNMT3B. Significantly, it was observed that following fucoidan treatment, there was an upregulation in both the mRNA and protein levels of MTSS1 (metastasis suppressor 1), a target gene known to be silenced by DNMT3B. Additionally, they revealed that fucoidan exhibited a down-regulatory effect on the TGF-β receptor and Smad signaling pathways in HCC cells. This down-regulation subsequently resulted in the inhibition of EMT, as evidenced by increased levels of E-cadherin and decreased levels of N-cadherin. Furthermore, fucoidan prevented extracellular matrix degradation by increasing TIMP-1 levels and decreasing MMP-2, MMP-9 levels. Ultimately, these molecular effects collectively diminished the invasive potential of HCC cells. The findings of their study provide compelling evidence regarding the significant impact exerted by fucoidan on the modulation of the miR-29b-DNMT3B-MTSS1 axis, as well as the suppression of TGF-β signaling in HCC cells. These results underscore the potential therapeutic value of fucoidan as a comprehensive treatment approach for addressing the invasive properties and metastatic tendencies associated with HCC [97].

#### NcRNA/DNMT3B axis modulation via nanostructured lipids-miRNA complex in cancer therapy

Nanostructured lipids (NLs), which were initially developed by Bangham in 1961 utilizing spherical vesicles, consist of a phospholipid bilayer measuring in the range of tens to hundreds of nanometers in diameter. NLs represent the pioneering nanoparticles employed in clinical medical investigations and have gained extensive utilization as carriers for a diverse range of small molecules, chemical compounds, and biopharmaceutical agents. Nanostructured lipid carriers (NLCs) exhibit enhanced entrapment efficiency, loading efficiency, and stability compared to other formulations [98]. At present, three primary categories of NLC have emerged, namely targeting-modified NLC, neutral NLC, and cationic NLC. Furthermore, these varieties of NLCs have garnered extensive application in the transportation of nucleic acids, encompassing various miRNA molecules employed in tumor gene therapy [99]. In recent investigations, there has been a notable emphasis on assessing the viability of cationic NLCs as potential vehicles for miRNA delivery in the context of lung cancer. Wu et al. postulated that reestablishing miR-29b levels in vivo, thereby targeting crucial genes involved in tumor initiation and progression, could potentially serve as a viable therapeutic approach for managing lung cancer. A carrier based on cationic lipoplexes (LPs) was devised, demonstrating efficient delivery of miR-29b in both in vitro and in vivo settings. They effectively administered miR-29b to NSCLC A549 cells via LPs referred to as LP-miR-29b. This delivery method exhibited notable competence in diminishing the levels of crucial targets such as cyclin-dependent protein kinase 6 (CDK6), DNMT3B, and myeloid cell leukemia sequence 1 (MCL1), consequently leading to a decline in cellular growth and clonogenicity in A549 cells. They observed a significant decrease in the IC50 of cisplatin within the cells treated with miR-29b. Additionally, in a murine xenograft model, LPs exhibited high efficiency in accumulating at specific tumor locations. They revealed that the administration of LP-miR-29b through systemic delivery resulted in a substantial fivefold elevation in the expression of miR-29b within the tumor. This approach effectively suppressed the mRNA expression of CDK6, DNMT3B, and MCL1 in the tumor tissue by approximately 57.4%, 40.5%, and 52.4% respectively. Furthermore, it significantly impeded tumor growth by approximately 60% compared to the negative control group receiving LP-miR-NC. Their findings provide compelling evidence to support the notion that cationic LPs serve as a highly effective delivery platform with immense prospects in the advancement of miRNA-focused therapeutic strategies for the treatment of lung cancer [100].

#### NcRNA/DNMT3B axis modulation via MAFMILHN in cancer therapy

In animal models, the application of nanoparticle-based delivery systems for anticancer drugs presents a compelling approach to amplify therapeutic benefits and diminish the occurrence of adverse effects. The disparity observed between preclinical data and clinical results is thought to arise from several factors, including the comparatively diminished impact of the enhanced permeability and retention (EPR) effect in human tumors, as well as the intrinsic or acquired resistance to monotherapy that can develop throughout the course of treatment. Additionally, the heterogeneity of human tumors further contributes to this discrepancy [101]. Consequently, there has been a shift in research attention towards the advancement of hybrid nanostructures composed of multiple materials. In this regard, Perepelyuk et al. assessed the therapeutic effectiveness and investigate the pharmacokinetics of hybrid nanoparticles (referred to as MAFMILHNs) loaded with miRNA-29b and functionalized with mucin1-aptamer in Severe combined immunodeficiency (SCID) mice bearing lung tumors. Initially, they reported a notable increase in apoptosis within the lungs of mice administered with MAFMILHNs compared to those treated with NC-nano and PBS. Their results illustrate that the tumor in experimental mice subjected to treatment with NC-nano (Control Nanoparticles) and PBS exhibited progressive growth throughout the duration of the study, whereas the tumor in mice treated with MAFMILHN (Magnetic and Fluorescent Multifunctional Inorganic Lipid Hybrid Nanoparticles) displayed a notable decrease in intensity over the same timeframe. The researchers successfully clarified the capacity of MAFMILHNs to suppress the oncoprotein DNMT3B both at the cellular and tissue levels. Furthermore, their findings indicate that MAFMILHN significantly outperformed negative control miRNA loaded hybrid nanoparticles (NC-nano) functionalized with MUC1-aptamer and lipofectamine-miRNA-29b (miRNA-29b lipo) in effectively down-regulating DNMT3B in A549 cells. Their results also illustrate the successful reduction of DNMT3B in lung tumors of SCID mice following treatment with MAFMILHN. In contrast, both NC-nano and PBS treatments did not exhibit significant effectiveness in reducing DNMT3B in the treated mice. They concluded that hybrid nanoparticles functionalized with MUC1-aptamer hold promising potential as a targeted nanoparticle delivery system for effectively delivering miRNA-29b to non-small cell lung cancer. This delivery system facilitates the downregulation of the target DNMT3B and ultimately leads to tumor suppression [102].

#### NcRNA/DNMT3B axis modulation via exosomes for cancer therapy

**Fig. 7:**
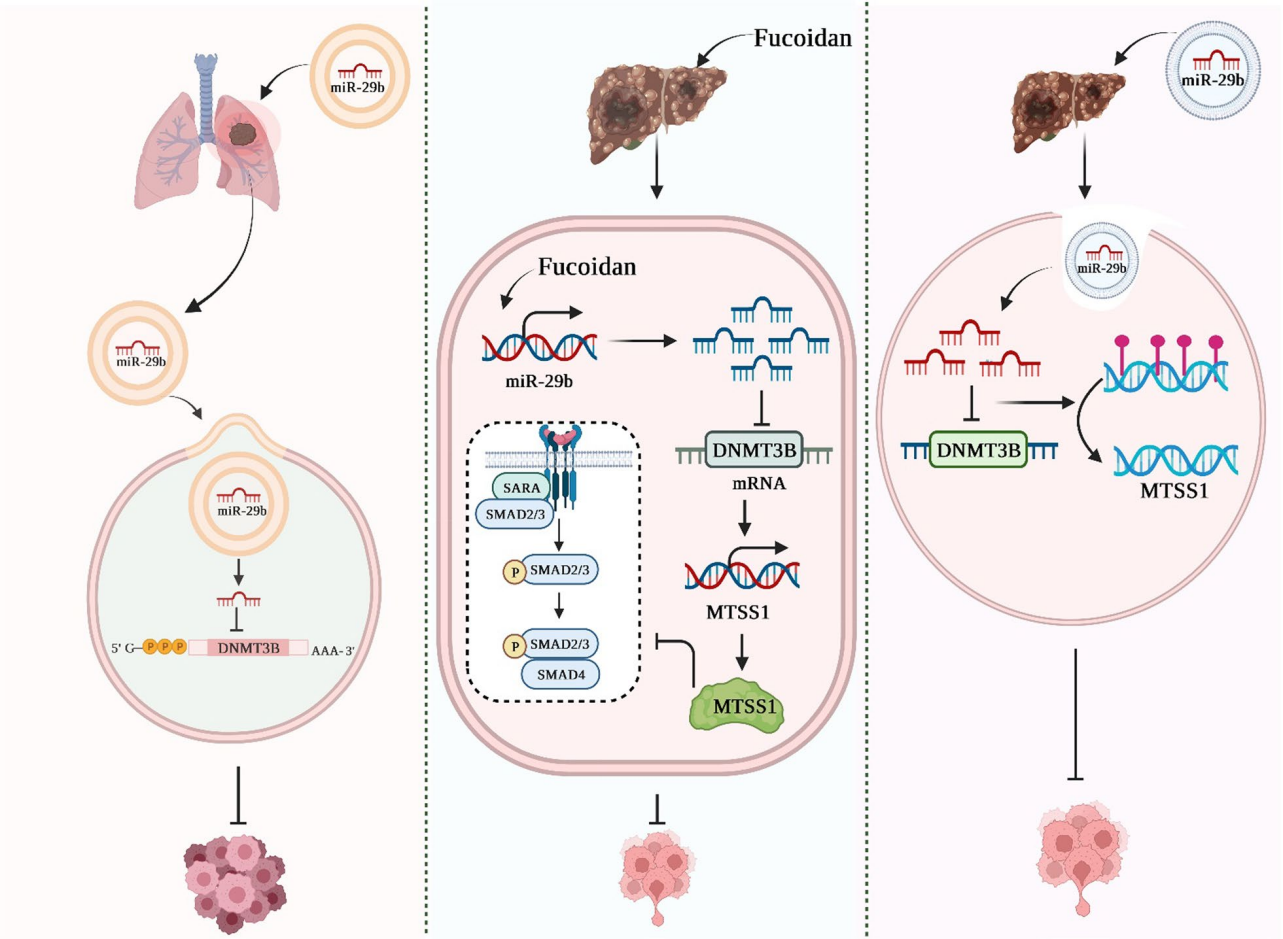
Targeting DNMT3B via ncRNAs in cancer therapy

Exosomes are a subtype of small extracellular vesicles (EVs) that originate from endosomes and have a diameter ranging from 40 to 150 nm. These vesicles are actively released by various cell types and can be found in numerous body fluids, such as breast milk, saliva, urine, cerebrospinal fluid (CSF), and blood. The amount of exosomes released and their composition can differ depending on factors like the manner in which they are generated, the specific cell type they originate from, and the physiological state of the cell. Extensive research has revealed that certain molecules exhibit high enrichment within exosomes, whereas others are found in minimal quantities. The potential clinical utility of exosomes has garnered increasing attention, particularly in the areas of disease biomarkers and therapeutic delivery systems. In this regard, Liu et al. explored the therapeutic potential of exosomal miR-29b derived from CAFs in HCC. During their investigation, they observed the direct transfer of miR-29b from CAFs to HCC cells through exosomes. They revealed that miR-29b exerts a direct inhibitory effect on the expression of DNMT3B in HCC cells. Additionally, they observed an upregulation of MTSS1 expression in these cells. Furthermore, they observed that these alterations led to growth arrest and hindered the invasion of HCC cells. Based on these findings, they concluded that exosomal miR-29b derived from CAFs plays a critical role in the development, progression, and metastasis of HCC. By functioning as a tumor suppressor that specifically targets DNMT3B, miR-29b holds promise as a potential therapeutic agent for HCC [103] (Fig. 7).

## Conclusion

Over the course of several decades, researchers have been actively involved in unraveling the underlying causes of human cancer, sparking intense discussions regarding the respective contributions of genetic and epigenetic abnormalities. The proliferation of data highlighting the significance of epigenetic mechanisms, particularly those leading to the suppression of crucial regulatory genes, has brought about an understanding that genetics and epigenetics synergistically function throughout all stages of cancer progression. DNA methylation plays a vital role as an epigenetic modification in the regulation of gene expression and has been closely linked to the initiation and progression of tumors. Within the DNA methyltransferase family, DNMT3B is a member that primarily operates in de novo methylation processes rather than the maintenance of existing methylation patterns. In the current work, we emphasized the reciprocal regulation between ncRNAs, including lncRNAs, circRNAs, and miRNAs, and DNMT3B, which is frequently overexpressed during cancer progression. These findings indicate that the interplay between ncRNAs and DNMT3B plays a crucial role in the pathogenesis of human cancers. Significantly, the ncRNA/DNMT3B axis, such as miR-145/DNMT3B interaction, emerges as a noteworthy prognostic factor in human cancer. Furthermore, we noticed that various therapeutic strategies, such as herbal medicine, Nanostructured lipids-miRNA complexes, MAFMILHN (Multifunctional Aptamer-Functionalized Magnetic Iron-Lanthanide Hybrid Nanoparticles), and exosomes through modulation of the ncRNA/DNMT3B axis, hold potential for implementation in cancer treatment.

## Data Availability

Not applicable.

## Abbreviations

DNMT3B: DNA methyltransferase 3 beta
ncRNAs: Non-coding RNAs
miRNA: MicroRNAs
lncRNAs: Long non-coding RNAs
circRNAs: Circular RNAs
DNMTs: DNA methyltranferases
3′-UTR: 3′-Untranslated region
RISC: RNA-induced silencing complex
GAS5: Growth arrest-specific transcript 5
MAP4K4: Mitogen-activated protein kinase 4
AGO: Argonaute
VIM: Vimentin
CRC: Colorectal cancer
CCND2: Cyclin D2
MSP: Methylation-specific PCR
ESCC: Esophageal squamous cell carcinoma
RNF2: Ring finger protein 2
EC: Esophageal carcinoma
GC: Gastric cancer
SKA3: Spindle and kinetochore associated complex subunit 3
OSCC: Oral squamous cell carcinoma
TSCC: Tongue squamous cell carcinoma
MEG3: Maternally expressed gene 3
LSCC: Laryngeal squamous cell carcinoma
EMT: Epithelial–mesenchymal transition
NSCLC: Non-small-cell lung cancer
NCAM1: Neural cell adhesion molecule 1
HIF-1α: Hypoxia-inducible factor-1α
GBM: Glioblastoma multiform
HCC: Hepatocellular carcinoma
LATS1: Large tumor suppressor gene 1
AML: Acute myeloid leukemia
CDCA3: Cell division cycle associated 3
HOTAIRM1: HOXA transcript antisense RNA myeloid-specific 1
CAFs: Cancer-associated fibroblasts
NFs: Normal fibroblasts
ECs: Endothelial cells
BC/BCa: Bladder cancer
HNF4γ: Hepatocyte nuclear factor 4 gamma
EPHA2: Erythropoietin-producing hepatocellular receptor tyrosine kinase class A2
ceRNA: Competing endogenous RNA
PC: Pancreatic cancer
PSCs: Pancreatic stellate cells
CCA: Cholangiocarcinoma
OS: Osteosarcoma
SOCS1: Suppressor of cytokine signalling 1
GAS7: Growth arrest specific 7
OvCa: Ovarian cancer
EOC: Epithelial ovarian cancer
PCa: Prostate cancer
CSCs: Cancer stem cells
BCSCs: Breast cancer stem cells
MTSS1: Metastasis suppressor 1
NLs: Nanostructured lipids
LPs: Lipoplexes
NLCs: Nanostructured lipid carriers
MCL1: Myeloid cell leukemia sequence 1
CDK6: Cyclin-dependent protein kinase 6
EPR: Enhanced permeability and retention
SCID: Severe combined immunodeficiency
IVIS: In vivo imaging system
miRNA-29b lipo: Lipofectamine-miRNA-29b
EVs: Extracellular vesicles
CSF: Cerebrospinal fluid
MAFMILHN: Multifunctional Aptamer-Functionalized Magnetic Iron-Lanthanide Hybrid Nanoparticles
